# A cutting-edge immunomodulatory interlinkage between HOTAIR and MALAT1 in tumor-associated macrophages in breast cancer: A personalized immunotherapeutic approach

**DOI:** 10.3389/fmolb.2022.1032517

**Published:** 2022-10-28

**Authors:** Hoda T. Amer, Reda A. Eissa, Hend M. El Tayebi

**Affiliations:** ^1^ Department of Pharmacology and Toxicology, The Molecular Pharmacology Research Group, Faculty of Pharmacy and Biotechnology, German University in Cairo, Cairo, Egypt; ^2^ Department of Surgery, Faculty of Medicine, Ain Shams University, Cairo, Egypt

**Keywords:** breast cancer, immunotherapy, epigenetics, tumor-associated macrophages (TAMs), MALAT1, HOTAIR, CD80, MSLN

## Abstract

Breast cancer (BC) is one of the most common cancers, accounting for 2.3 million cases worldwide. BC can be molecularly subclassified into luminal A, luminal B HER2-, luminal B HER2+, HER2+, and triple-negative breast cancer (TNBC). These molecular subtypes differ in their prognosis and treatment strategies; thus, understanding the tumor microenvironment (TME) of BC could lead to new potential treatment strategies. The TME hosts a population of cells that act as antitumorigenic such as tumor-associated eosinophils or pro-tumorigenic such as cancer-associated fibroblasts (CAFs), tumor-associated neutrophils (TANs), monocytic-derived populations such as MDSCs, or most importantly “tumor-associated macrophages (TAMs),” which are derived from CD14^+^ monocytes. TAMs are reported to have the pro-inflammatory phenotype M1, which is found only in the very early stages of tumor and is not correlated with progression; however, the M2 phenotype is anti-inflammatory that is correlated with tumor progression and metastasis. The current study focused on controlling the anti-inflammatory activity in TAMs of hormonal, HER2+, and TNBC by epigenetic fine-tuning of two immunomodulatory proteins, namely, CD80 and mesothelin (MSLN), which are known to be overexpressed in BC with pro-tumorigenic activity. Long non-coding RNAs are crucial key players in tumor progression whether acting as oncogenic or tumor suppressors. We focused on the regulatory role of MALAT1 and HOTAIR lncRNAs and their role in controlling the tumorigenic activity of TAMs. This study observed the impact of manipulation of MALAT1 and HOTAIR on the expression of both CD80 and MSLN in TAMs of BC. Moreover, we analyzed the interlinkage between HOTAIR and MALAT1 as regulators to one another in TAMs of BC. The current study reported an upstream regulatory effect of HOTAIR on MALAT1. Moreover, our results showed a promising use of MALAT1 and HOTAIR in regulating oncogenic immune-modulatory proteins MSLN and CD80 in TAMs of HER2+ and TNBC. The downregulation of MALAT1 and HOTAIR resulted in the upregulation of CD80 and MSLN, which indicates that they might have a cell-specific activity in TAMs. These data shed light on novel key players affecting the anti-inflammatory activity of TAMs as a possible therapeutic target in HER2+ and TNBC.

## Introduction

Breast cancer (BC) is one of the most commonly diagnosed cancers in women. It has now exceeded lung cancer as the leading cause of overall cancer incidence in 2020, with 2.3 million new cases; 685,000 deaths occur due to BC, making it the fifth leading cause of cancer mortality worldwide (Sung et al., 2021). Due to the molecular heterogeneity, the subtypes of BC are divided according to the expression of estrogen receptor (ER), progesterone receptor (PR), and human epidermal growth factor receptor 2 (HER2), in addition to the percentage of the proliferating index (Ki67). Accordingly, they are classified into five different subtypes: luminal A, luminal B HER2-, luminal B HER2+, HER2+, and TNBC (basal-like) (Kondov et al., 2018). The luminal A subtype is considered the most abundant subtype with 50–60% prevalence among BC patients and is characterized by the expression of ER and/or PR with no expression of HER2 and with Ki67 < 14% (Blows et al., 2010), while luminal B abundance is 10–20% among BC patients with a more aggressive diagnostic profile than luminal A and is further classified into luminal B HER2- and luminal B HER2+. The luminal B HER2- subtype is characterized by the expression of ER and/or PR with no expression of HER2 and with Ki67 ≥ 14% (Blows et al., 2010), while the luminal B HER2+ subtype expresses ER and/or PR+ in addition to HER2+ with the expression of any Ki67 percentage (Blows et al., 2010). The HER2-enriched subtype is hormonal negative, expressing only HER2 receptors with any Ki67 percentage. The HER2-enriched subtype suffers from a worse prognosis than luminals in spite of the availability of its targeted therapy, anti-HER2. Finally, TNBC, ER, PR, and HER2- have the worst prognosis of all subgroups (Blows et al., 2010).

Generally, the immune system plays a major role in cancers. Immunity can be subcategorized into innate immunity, which is fast, immediate, and non-specific, and adaptive immunity, which is specific and long lasting (Wills-Karp, 2007). Within innate immunity, myeloid cells are the most abundant hematopoietic cells (Gabrilovich et al., 2012). Tumor-infiltrating myeloid cells include granulocytes (such as basophils, eosinophils, and neutrophils), monocytes, dendritic cells, tumor-associated macrophages (TAMs), immature myeloid cells (IMCs), and MDSCs (6). Recently, the tumor-infiltrating myeloid cells have been reported to have an important role in angiogenesis, invasion, and metastasis, indicating their possible immunosuppressive role (Gabrilovich et al., 2012).

Tumors have the ability to recruit stromal cells (e.g., fibroblasts), immune cells, and vascular cells through the secretion of growth factors, cytokines, and chemokines building a tumor microenvironment (TME) by releasing growth-promoting signals and remodeling tissue structure affecting initiation, progression, metastasis, vascularization, and therapy responses (Garofalo et al., 2006). Many treatments focus only on the cancer cell while special attention to the TME is needed since it has the key players of BC development and progression. Tumor not only tries to escape from the host immune system but also benefits from the infiltrating cells by modifying their functions to create the microenvironment that is favorable to its progression (Pollard, 2004).

Monocytes play a very critical role in the TME either by themselves or on reprogramming to myeloid-derived suppressor cells (MDSCs) or tumor-associated macrophages (TAMs) (Allaoui et al., 2016). Monocytes are divided into three subsets based on the expression of CD16 and CD14 surface markers (Coillard and Segura, 2019). The CD16 (FcgRIII) molecule was only known initially to be expressed on mature macrophages; however, it is recognized recently as a surface marker for monocytes (Feng et al., 2011). The three subsets of monocytes are “classical” CD14^+^CD16^−^ monocytes, which make up around 85% of monocytes, “intermediate” CD14^+^CD16^+^ monocytes, which account for 5–10% of total monocytes, and finally, “non-classical” CD14^−^CD16^+^, which also accounts for 5–10% (Coillard and Segura, 2019).

Within the TME, monocytes can differentiate to M1 macrophage, which expresses the CD163 receptor marker at low levels (CD163neg/low macrophages), mediating defensive immune response (Ding et al., 2016). M1 macrophages usually receive stimulation from GM-CSF, LPS, and IFN- γ to produce IL-23 and IL-12 and promote Th1 responses (Rey-Giraud et al., 2012), thus having a pro-inflammatory response. In addition, M1 can secrete IL-6, ROS, and TNF- α (Sousa et al., 2015). Moreover, monocytes can infiltrate the tumor and differentiate into the M2 subtype, which is CD163 high (Feng et al., 2011). M2 can reduce tissue damage caused by inflammatory processes and stimulate their repair, thus having anti-inflammatory responses. M2 is usually activated by M-CSF, IL-4, IL-10, and IL-13 and can produce anti-inflammatory IL-10 and TGF-β. In the presence of tumors, macrophages are plastic and can be reprogrammed to polarize into either M1-like macrophages or M2-like macrophages, according to the cytokines present in the TME (Benner et al., 2019). Interestingly, TAMs contribute 5–40% of tumor mass in solid tumors. In the beginning, TAMs are said to have a pro-inflammatory phenotype (M1-like) and inhibit the tumorigenesis by ROS and TNF- α or even phagocytosis (Zhou et al., 2020). Nevertheless, once the cancer starts progressing, TAMs tend to secrete IL-10, TGF-β, and IL-12, suppressing cytotoxic T lymphocyte (CTL) and NK cells with the upregulation of programmed death ligand-1 (PDL-1), thus having anti-inflammatory action (Zhou et al., 2020). Moreover, TAMs can induce Tregs activity by IL-10, TGF- β, and PDGF-2, thus suppressing T cells. TAMs also promote angiogenesis by releasing VEGF, PDGF, and IL-8. Furthermore, they contribute to invasion and metastasis, particularly in malignant solid tumors, reducing the survival in BC patients and worsening their clinical outcomes (Campbell et al., 2011). TAMs contribute to the extracellular tissue remodeling, thus metastasis *via* MMPs. It was observed that COX-2 in breast TAMs promotes the metastatic potential of breast cancer cells. COX-2 in TAMs induces MMP-9 expression and enhances epithelial–mesenchymal transition (EMT) in breast cancer cells (Gan et al., 2016). Not only this but also TAMs express VEGF-C/D at the tumor site, which is shown to be related to lymph node metastasis (Schoppmann et al., 2006). TAMs not only play a role in BC but also in other cancers, namely, colorectal cancer. A study was carried out to show the contribution of TAMs in the metastasis of colorectal cancer (CRC). Briefly, the study showed that CRC-conditioned macrophages regulated EMT to enhance migration and invasion by the secretion of IL-6. TAMs-derived IL-6 was shown to activate the JAK2/STAT3 pathway. STAT3 transcriptionally inhibited the tumor suppressor miR-506-3p in CRC cells (Wei et al., 2019). It was also shown in another study that IL-6 secreted by TAMs promote the invasion of the prostate cancer cells and express low levels of the epigenetic factor (SFMBT2) (Gwak et al., 2020).

In general, immune cells are known to express a number of immunomodulatory proteins, including CD80 and mesothelin (MSLN) proteins. CD80 (cluster of differentiation 80), a type-1 transmembrane glycoprotein is first identified in Epstein–Barr virus-activated B-cell blasts, B lymphoblastoid cell lines, and Burkitt’s lymphomas (Mir, 2015). It is a costimulatory molecule known to activate T cell and regulate the activity of normal and malignant B cells (Orabona et al., 2004). CD80 is expressed on activated B cells, macrophages, DCs, and cancer cells and binds to CD28 on T cells. It was shown that the loss of CD80 is enough to allow tumor to escape the immune system, thus imparting apoptosis caused by tumor-infiltrating T cells (Lázár-Molnár et al., 2010). Consequently, even if the tumor cell expresses MHC-I molecule while the co-stimulation is absent, the recognition of antigens by CTL cells will not cause any response. Conversely, on transfecting tumor cells with CD80, the tumor cell is found to be more susceptible to the lysis by T cells *ex vivo*. Moreover, it was evident that CD80 may enhance the memory responses by CTLs (Lázár-Molnár et al., 2010). However, CD80 has also been reported to be overexpressed in a number of cancers including BC (Li et al., 2020). This may be explained by the fact that CD80 is a ligand not only for CD28 but also for CTLA4 (cytotoxic T lymphocyte antigen-4 or CD152). CTLA4 displays important sequence and structure homology with CD28. Conversely to CD28, CTLA4, a negative regulator of T-cell activation, was found to have an anti-inflammatory action and facilitated the escape of tumor immunity (Brahmer et al., 2015). In BC, it was reported that CTLA4 has a higher binding affinity to CD80 than CD28 (Walker and Sansom, 2011). Consequently, blocking CTLA4 is a target for immunotherapy (Walker and Sansom, 2011). As a matter of fact, CD80 is highly expressed on APCs including macrophages, thus it is expected to be highly expressed on TAMs. Upon isolation of TAMs from human renal cell carcinomas, TAMs were shown to induce the CTLA4 expression on T lymphocytes (Daurkin et al., 2011).

Mesothelin (MSLN) is another immunomodulatory protein that is present on normal mesothelial cells of the pleura, peritoneum, and pericardium (Hassan et al., 2004). The MSLN gene is shown to be overexpressed in many cancers including ovarian cancer, adenocarcinoma, pancreatic cancer, mesothelioma, lung adenocarcinoma, acute myeloid leukemia (Pastan et al., 1992), endometrial adenocarcinomas, and squamous cell carcinomas of the cervix, lung, head, and neck (Ordóñez, 2003). MSLN is reported to be overexpressed in 67% of TNBC cases and less than 5% of hormonal BC. Not only this but also the presence of MSLN in BC cells is associated with tumor infiltration of the lymph node. MSLN is shown to have a very limited expression in normal tissues, thus making it a very attractive candidate for cancer therapy (Macdonald et al., 2016).

Several immunomodulatory proteins are known to be controlled epigenetically. Basically, the epigenetic machinery is composed of three components, namely, DNA methylation, histone modification, and non-coding RNAs. Non-coding RNAs (ncRNAs) are classified into two major classes based on the transcript size, in which 200 nucleotides is the threshold: small ncRNAs (sncRNAs) and long non-coding RNAs (lncRNAs) (Carninci et al., 2005).

Metastasis associated in lung adenocarcinoma transcript 1 (MALAT1) is considered as one of the most studied lncRNAs in cancer and is also known as nuclear enrichment autosomal transcript 2 (NEAT2). MALAT1 was observed to be highly expressed in cancer tissues and it was initially observed in the metastatic non-small lung cancer tissues (Guan et al., 2020). Its function is still controversial that whether it acts as oncogenic or tumor suppressor lncRNA, thus understanding MALAT1 better would be important in epigenetics studies. One mechanism of how MALAT1 functions as an oncogene is by interacting with the polycomb repressive complex 2 (PRC2). On interaction, the RNA–protein complex with EZH2 and SUZ12 is formed. EZH2 and SUZ12 are two components of the PRC2 complex, thus facilitating the histone H3K27 trimethylation at the promoters of some tumor suppressor genes such as E-cadherin and N-myc downregulated gene-1 (NDRG1). Consequently, b-catenin and c-myc expression are increased (Chen et al., 2020). On the other hand, MALAT1 can function as a tumor suppressor since its expression was found to be positively correlated with the expression of the tumor suppressor PTEN, and their decreased levels were associated with mortality and poor patient survival in both colorectal cancer and BC patients (Guan et al., 2020).

MALAT1 is shown in the literature to have an oncogenic activity enhancing both the progression and metastasis of breast tumors. A research group studied this oncogenic potential of MALAT1 by using MTT and transwell assay to detect proliferation, migration, and invasion. Furthermore, the drug resistance test was performed to assess the sensitivity of BC cells to doxorubicin. The study has shown that silencing of MALAT1 could significantly suppress the proliferation, migration, and invasion of BC cells. Moreover, downregulation of MALAT1 sensitized BC cells to doxorubicin (Yue et al., 2021). Not only this but also another group used the MMTV (mouse mammary tumor virus)-PyMT mouse mammary carcinoma model to assess the proliferative and metastatic potential of MALAT1. The results showed slower tumor growth accompanied by significant differentiation into cystic tumors and a reduction in metastasis upon loss of MALAT1 (Arun et al., 2016).

In addition, HOTAIR (HOX transcript antisense RNA) is the first lncRNA found to promote tumor progression. HOTAIR is highly expressed in metastatic BC. It is transcribed from the antisense strand of the HOXC genes and partly overlaps with HOXC11 (Rinn et al., 2007). HOTAIR can function as an oncogene using several mechanisms, especially by the negative regulation of a number of miRNAs. For example, it competes with miR-34a, leading to the upregulation of SOX2, thus cell proliferation. It can also promote cell growth, mobility, and invasiveness by suppressing miR-20a-5p and consequently upregulate HMGA2 (Li et al., 2016).

HOTAIR is known in the literature to have a proliferative and metastatic potential. In a research study, CCK-8 and colony formation assays showed that HOTAIR overexpression promoted the proliferation of MCF-7 cells. Furthermore, transwell invasion and migration assays showed that HOTAIR overexpression increased the migration and invasion of BC cells. These results indicate that HOTAIR facilitates the growth and metastasis of BC cells *in vitro* (He et al., 2022). Another research study confirmed these findings by using qRT-PCR to determine the expression of HOTAIR. CCK-8 and transwell assays were also used to detect the proliferation, migration, and invasion of cells. In addition, animal experiments were conducted to validate the effect of HOTAIR on BC tumor growth *in vivo*. The results showed that HOTAIR was upregulated in BC tissues and cells, and its knockdown suppressed the proliferation, migration, invasion, and the activity of the AKT signaling pathway of BC cells. Additionally, interference of HOTAIR had impacted BC tumor growth *in vivo* (Wang et al., 2020).

Even though TAMs have a crucial role in tumorigenesis, the epigenetic players controlling and regulating this anti-inflammatory activity have never been studied before through their direct manipulation in TAMs. The aim of this study is to investigate the interlinkage between MALAT1 and HOTAIR and their regulatory activity on immunomodulation by examining the expression profile of CD80 and MSLN in tumor-associated macrophages in the hormonal, HER2+, and TNBC subtypes.

## Materials and methods

### Blood sample collection and preparation

In total, 43 blood samples were collected in EDTA tubes from breast cancer (BC) patients, after taking their consents. All patients were females ranging between the age of 34 and 78 years. Clinical features for each patient are represented in Table 1. Gender-matched healthy controls were used. Within 3 or 4 h of sample collection, the Ficoll separation technique was optimized and used to isolate the PBMCs from the whole blood (Jaatinen and Laine, 2007). The PBMCs of each sample were cryopreserved and stored in the −80 C freezer for later use. Detailed clinical features for each patient are mentioned in Supplementary Table S1.

**TABLE 1:** Clinical features of collected BC patients.

Patient, *n*	Percentage
Age	
Less than 50 (*n* = 15/43)	34.8%
More than 50 (*n* = 28/43)	65.11%
Molecular subtype	
Hormonal (23/43)	53.4%
HER2+ (*n* = 10/43)	23.2%
TNBC (*n* = 10/43)	23.2%
Histological classification	
Invasive ductal carcinoma (*n* = 39/43)	90.6%
Invasive lobular carcinoma (*n* = 3/43)	6.9%
Inflammatory (*n* = 1/43)	2.3%
Mammogram examination	
BIRADS 4a (*n* = 1/43)	2.3%
BIRADS 4b (*n* = 1/43)	2.3%
BIRADS 4c (*n* = 4/43)	9.3%
BIRADS 5 (*n* = 10/43)	23.2%
BIRADS 5 (*n* = 27/43)	62.7%
Treatment	
Chemotherapy (*n* = 4/43)	9.3%
Chemotherapy + anti-HER2 treatment (*n* = 6/43)	13.9%
No neoadjuvant chemotherapy (*n* = 32/43)	74.41%
Hormonal therapy (*n* = 1/43)	2.3%

### Cell culture

Cryopreserved PBMCs are left to melt at room temperature. The melted PBMCs are transferred into a falcon tube containing a 6 ml wash mix and was allowed to shake for 5 min followed by centrifugation for 5 min at 1,500 rpm. A pellet of PBMCs is formed, and the supernatant is discarded. The cells are suspended in an appropriate volume of complete media and counted.

### CD14^+^ monocyte’s isolation

Isolation of CD14^+^ monocytes by negative depletion was performed using MojoSort™ Human CD14^+^ Monocytes Isolation Kit protocol (Cat. No. 480047), MojoSort™ Buffer (5X) (Cat. No. 480017), and MojoSort™ Magnet (Cat. No. 480019/480020).

### CD8^+^ T-cell isolation

Isolation of CD8^+^ T cells by negative depletion was carried out using MojoSort™ Human CD8^+^ T cell Isolation Kit protocol (Cat. No. 480012), MojoSort™ Buffer (5X) (Cat. No. 480017), and MojoSort™ Magnet (Cat. No. 480019/480020).

### CD14^+^ monocyte differentiation to TAMs

Freshly isolated CD14^+^ monocytes were subjected to centrifugation at 1,500 rpm, buffer supernatant was discarded, and CD14^+^ monocytes were plated in a 48-well plate (10,000 cells per well). Monocytes were cultured in 1:1 ratio of 10% cell culture medium and TCM with the addition ofIL-4 (1 μg/ml) (Schenendoah (United States), ID:100-09), IL-10 (1 μg/ml) (Schenendoah (United States), ID:100-83), and M-CSF (1 μg/ml) (Schenendoah (United States) 100-03). The medium was refreshed every other day. TAMs were harvested on day 7.

### Transfection of TAMs by the lipofection method of nucleic acid delivery

After incubation of TAMs for 7 days to ensure differentiation (3 × 10^4^ cells per well in a 48-well plate), the supernatant was discarded and refreshed by the transfection media. The appropriate amount of siRNAs (2 ul) was diluted in 60 μL of free culture medium without serum. The appropriate amount of HiPerFect transfection reagent (Qiagen) was added (1 ul) to the diluted siRNAs (MALAT1 siRNA, Qiagen ID: SI04342233) (HOTAIR siRNA, Qiagen ID: SI04446036) and then mixed by vortexing. The siRNAs-HPTR mixture was incubated for 5–10 min at room temperature (15–25°C) to allow the formation of transfection complexes. The complex was then added dropwise onto the cells. The plates were then rotated gently to ensure uniform distribution of the transfection complexes. After 6 h, 140 ul complete culture media of RPMI containing serum and antibiotics were added to the PBMCs and incubated for 48 h for RNA extraction and subsequent analysis of gene silencing or induction.

### Total RNA isolation from TAMs

Total RNA was isolated from TAMs, which were previously differentiated from CD14^+^ monocytes and treated against diluted silencers. Total RNA was extracted using Thermo Scientific GeneJET RNA Purification kit (Catalog no: K0732), according to the manufacturer’s protocol. In brief, lysis buffer and absolute ethanol were added to each sample eppendorf and centrifuged for 1 min at 12,000 × g. The flow-through solution in the collection tube was then discarded. Each sample was washed twice with wash buffer 1 and wash buffer 2. Finally, the collection tube containing the flow-through solution was discarded, and the GeneJet RNA purification column was transferred to a sterile 1.5-ml RNAse-free microcentrifuge tube. Nuclease-free water was finally added, and the centrifugation was repeated. Purified RNA was stored in a −80°C freezer.

### Reverse transcription of total mRNA into total cDNA

Total RNA extracted was reverse transcribed into the single-stranded cDNA using the high-capacity cDNA reverse transcription kit (Thermo Fisher, Cat No: K1652), according to the manufacturer’s protocol. Each component of the reverse transcription kit and the extracted RNA of each sample were thawed on ice and mixed by vortexing to ensure appropriate resuspension. Each reaction’s total tube volume was 20 ul with 1:1 ratio (reaction mix: total RNA). Finally, the reaction tubes were placed in a thermo cycler with a heated lid whose thermal profile was adjusted, according to the manufacturer’s protocol. All the cDNA samples were stored in the −20°C freezer until qRT-PCR analysis was performed.

### Quantitative analysis of target genes

The expressions of MALAT1, HOTAIR, CD80, and MSLN mRNA levels were quantified using RT-PCR. Reagents used were the TaqMan, MALAT1, HOTAIR, CD80, and MSLN expression assays (Themo Fisher (United States)-TaqMan MALAT1 assay (Hs00273907), TaqMan CD80 assay (Hs01045161_m1), TaqMan HOTAIR assay (Hs05502358_s1), and TaqMan MSLN assay (ID: Hs00245879) along with B-actin as an endogenous control housekeeping gene to normalize the expression values. Probes used for MALAT1, HOTAIR, CD80, and MSLN were labeled with the FAM reporter dye. B-actin was reported with the VIC reporter dye. Each reaction tube’s total volume was 20 ul with 1:4 ratio (total cDNA:reaction mix). Each reaction mix was composed of nuclease-free water, Premix Ex TaqTM (Probe qPCR), TaqMan target gene assay expression assay (x20), and B-actin (VIC).

### StepOne® real-time PCR results and interpretation

Upon preparing the reaction tubes, they were placed into the StepOne® real-time PCR instrument and the run was performed in the standard mode, consisting of two stages. A first stage where the Taq-polymerase enzyme is activated followed by the second stage of 40 amplification cycles (each cycle comprises a 15 second denaturation step followed by 60 s of annealing and extension). The StepOne® real-time PCR yields a cycle threshold value (Ct) for each sample, which represents the fractional cycle number at which the fluorescence produced exceeds a threshold line. Each cycle threshold (Ct) obtained was subsequently used for quantification of the amplified target compared to its endogenous control housekeeping gene (B-actin for target genes), yielding a ΔCt value.

### Protein quantification of VEGF-A in TAM culture media using ELISA

After transfecting TAMs with silencers against MALAT1 and HOTAIR, VEGF-A protein was quantitatively examined using the VEGF-A Human ELISA Kit (Invitrogen, BMS277-2). The kit is a sandwich ELISA, which is designed to measure the amount of the target bound between a matched antibody pair. Briefly, 400 μL wash buffer per well was used to wash pre-coated microwells from the microplate. After washing, different concentrations of standards were added to the wells, according to the manufacturer’s protocol. The plates were incubated for 2 h at room temperature. After washing, 100 μL of biotin-conjugated antihuman VEGF-A polyclonal antibody (1:100) was then added to the wells and incubated for 1 h at room temperature. After washing, 100 μL of horseradish peroxidase-labeled streptavidin was added to the wells and incubated for 1 h at room temperature. After washing, 100 µL of TMB substrate solution was added to all wells. Color development on the plate is monitored for 30 min at room temperature, then the stop solution was added to terminate the reaction, and the absorbance was analyzed for each microwell for both standards and samples at 450 nm wavelength. The results are calculated by constructing a standard curve plotting the mean OD and concentration for each standard.

### MDA-MB-231 cell line culturing

MDA-MB-231 cell line was purchased from the tissue culture unit, Egyptian Company for Vaccines and Sera, after proper authentication and testing for *mycoplasma* contamination. MDA-MB-231 cells are ER, PR, and HER2-. MDA-MB-231 cells were cultured using our previous protocol (Hamed et al., 2021). The cell line was cultured in a suitable culture media [high-glucose DMEM supplemented with 10% FBS and 1% penicillin/streptomycin in 10 cm Petri dishes (Gibco, United States)]. Culture media were changed every 2–3 days until the cells reached 80–90% confluency. The cells were washed with PBS, trypsinized, and split into two clean Petri dishes. After the second splitting, the cells were harvested and prepared for further experimentation. The cells were kept in a 37°C, 5% CO_2_ incubator. Prior to seeding and experimentation, the cells were counted and checked for viability using trypan blue.

### CD8^+^ T-cell culture in the TAM supernatant

In a 96-well plate, CD8^+^ T cells were cultured (2-3 x 104 cells per well) in 200 μL culture media (100 μL of TAMs supernatant +100 μL of full RPMI media) for 24 h. The supernatant of TAMs culture media was isolated after TAM culturing for 7 days under four conditions (untransfected TAMS, siMALAT1 TAMs, siHOTAIR TAMs, and siMALAT1/HOTAIR TAMs).

### LDH cytotoxicity assay

After culturing CD8^+^ T cells in TAM-conditioned media, LDH toxicity assay was performed to evaluate the cytotoxicity potential of CD8^+^ T cells upon co-culturing with MDA-MB-231 cell line with and without the addition of PDL-1 inhibitor drug. LDH assay is used as rapid determination of cytotoxicity based on lactate dehydrogenase released into the cell culture medium using the Canvax Biotech protocol (Cat No: CA0020). In the LDH cytotoxicity assay, the lysis control wells are first prepared with the addition of lysis solution and incubating the plate in a 37°C, 5% CO_2_ incubator for 45 min. Then, 50 μL of culture supernatant from each well is transferred to a new 96-well flat-bottom plate and the reaction mixture is then added (50 μL on each well). After incubation for 30 min, the reaction is terminated by the addition of stop solution (50 μL on each well). Absorbance for all controls and experimental samples is measured at 450 nm wavelength. Data are analyzed by measuring the % relative cytotoxicity.

### Flow cytometry

To evaluate the CD8^+^ T-cell isolation efficiency using flow cytometry [CytoFLEX, Beckman Coulter Life Sciences (United States)], CD8^+^ was measured using flow cytometry anti-CD8 PE (Biolegend, Cat No: 344706). In brief, the cells were first dissociated, and then, single-cell suspensions were prepared (240,000 cell/tube). The cells were then washed twice with 2 mls (PBS 1% FBS) and centrifuged at 350 × g for 5 min. After washing, 1.2 µg anti-CD8 PE was added (5 µg per one million cells), and the cells were incubated at 4° for 30 min.

### Statistical analysis

All data were expressed in relative quantitation (RQ) for RT-qPCR. For the purpose of comparison between two different studied groups, Student’s unpaired *t*-test was used. One-way Anova followed by Dunette’s test of multiple comparison was used for the comparison between more than two different studied groups. Data were expressed as mean ± standard error of the mean (SEM). A *p*-value less than 0.05 were considered statistically significant **** = *p* < 0.0001, *** = *p* < 0.001, ** = *p* < 0.01, and * = *p* < 0.05. Analysis was performed using GraphPad Prism 7.02 software.

## Results

### Expression profiling of LncRNAs MALAT1 and HOTAIR in BC TAMs

**FIGURE 1:**
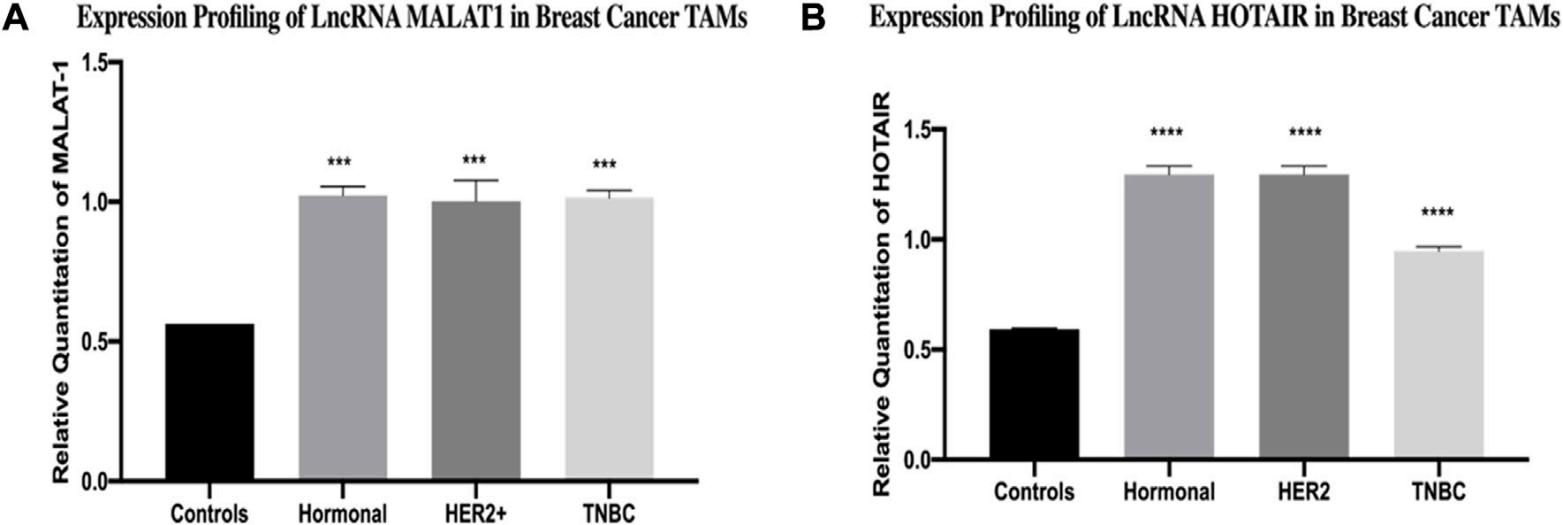
Expression profiling of LncRNA MALAT1 and HOTAIR in breast cancer TAMs. q-PCR was performed for the quantitative analysis and expression profiling of the MALAT1 and HOTAIR levels in BC subtypes (hormonal, HER2+, and TNBC) compared to healthy donors. The results showed significant upregulation of **(A)** MALAT1 and **(B)** HOTAIR in hormonal, HER2+, and TNBC compared to healthy donors. One-way Anova followed by Dunette’s test of multiple comparison was used for the purpose of comparison between more than two different studied groups. **** = *p* < 0.0001, *** = *p* < 0.001, ** = *p* < 0.01, * = *p* < 0.05, and ns = statistically not significant.

The expression of MALAT1 was found to be upregulated in TAMs of the hormonal, HER2+, and TNBC compared to healthy donors (*p* = 0.0002, 0.0001, and 0.0001, respectively). In a similar pattern, the expression of HOTAIR was found to be upregulated in TAMs of the three subgroups (hormonal, HER2+, and TNBC) compared to healthy donors (*p*=<0.0001, <0.0001, and <0.0001, respectively), as shown in Figure 1.

### Expression profiling of CD80 and MSLN in breast cancer TAMs

**FIGURE 2:**
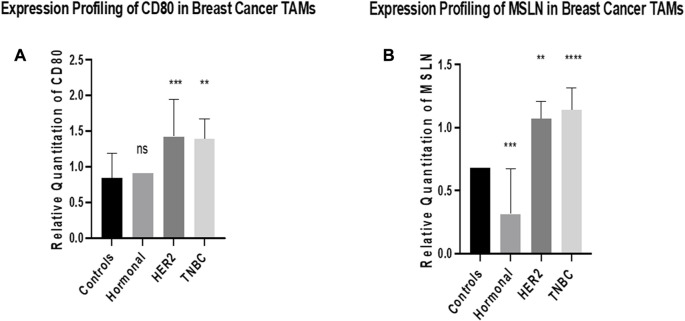
Expression profiling of CD80 and MSLN in breast cancer TAMs. q-PCR was performed for the quantitative analysis and expression profiling of the CD80 and MSLN levels in TAMs of BC subtypes (hormonal, HER2+, and TNBC) compared to healthy donors. **(A)** Results showed a non-significant change in the expression profile between TAMs of the hormonal subtype and healthy donors. Conversely, a significant upregulation of CD80 was observed in TAMs of HER2+ and TNBC compared to healthy donors. **(B)** Expression of MSLN showed significant downregulation in TAMs of the hormonal subtype compared to healthy donors. Conversely, a significant upregulation of MSLN was observed in TAMs of HER2+ and TNBC compared to healthy donors. One-way Anova followed by Dunette’s Test of multiple comparison was used for the purpose of comparison between more than two different studied groups. **** = *p* < 0.0001, *** = *p* < 0.001, ** = *p* < 0.01, * = *p* < 0.05, and ns = statistically not significant.

The expression of CD80 was found to have a non-significant difference in TAMs of the hormonal subtype compared to healthy donors. However, it was significantly upregulated in TAMs of both HER2+ and TNBC subtypes (*p* = 0.0008 and 0.0016 respectively) compared to TAMs of healthy donors. Meanwhile, the expression of MSLN was found to have significant downregulation in TAMs of the hormonal subtype (*p* = 0.0002) compared to healthy donors. However, it was significantly upregulated in TAMs of both HER2+ and TNBC subtypes (*p* = 0.0023 and <0.0001, respectively) compared to TAMs of healthy donors, as shown in Figure 2.

### HOTAIR and MALAT1 silencing efficiency in TAMs of BC

**FIGURE 3:**
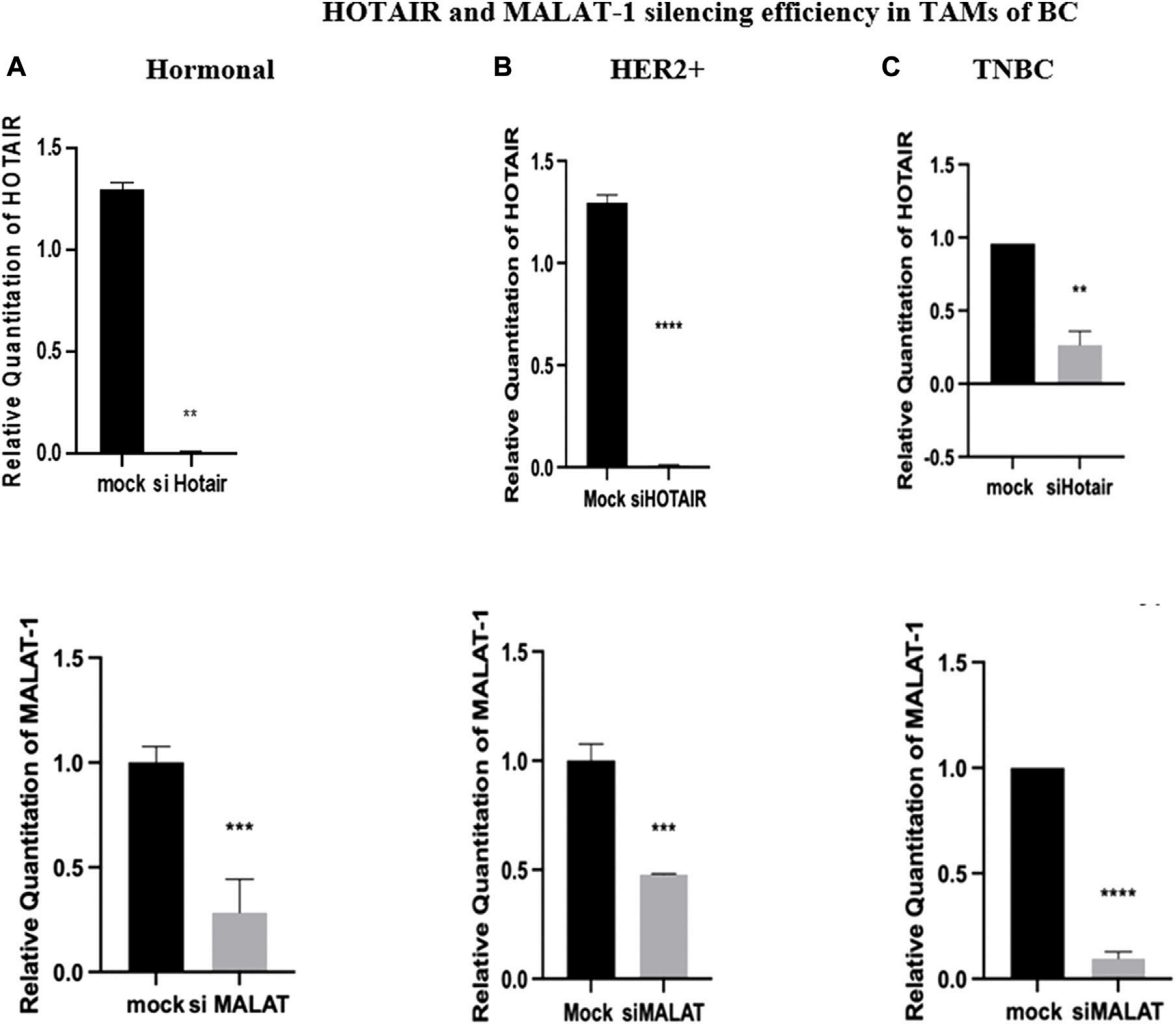
HOTAIR and MALAT1 silencing efficiency in TAMs of **(A)** hormonal, **(B)** HER2+, and **(C)** TNBC. q-PCR was performed for the quantitative analysis of the HOTAIR and MALAT1 levels. Transfection with silencers against HOTAIR resulted in a decrease in expression with a rate of approximately 1.29 folds in both hormonal and HER2+, while a decrease in expression with a rate of approximately 0.75 folds in TNBC compared to the normal untransfected controls (mocks). With a similar pattern of expression, transfection with silencers against MALAT1 resulted in a decrease in expression with a rate of approximately 0.8 folds in hormonal, 0.5 folds in HER2+, and 0.9 folds in TNBC compared to the normal untransfected controls (mocks). Thus, the efficiency of silencing of HOTAIR and MALAT1 was confirmed. Parametric student *t*-test was used for comparing two groups. **** = *p* < 0.0001, *** = *p* < 0.001, ** = *p* < 0.01, * = *p* < 0.05, and ns = statistically not significant.

For transfection efficiency purposes, HOTAIR and MALAT1 expressions were analyzed after transfection with their silencers (siRNAs). The transfection of TAMs with silencers against HOTAIR resulted in downregulation, thus silencing the expression of HOTAIR in hormonal, HER2+, and TNBC (*p* = 0.0010, <0.0001, and 0.0059, respectively) in comparison to untransfected controls (mocks). Moreover, the transfection of the TAMs with silencers against MALAT1 resulted in downregulation, thus silencing of the expression of MALAT1 in hormonal, HER2+, and TNBC (*p* = 0.0009, 0.0008, and <0.0001 respectively), as shown in Figure 3.

### Impact of HOTAIR silencing on MALAT1 expression in TAMs of BC patients

**FIGURE 4:**
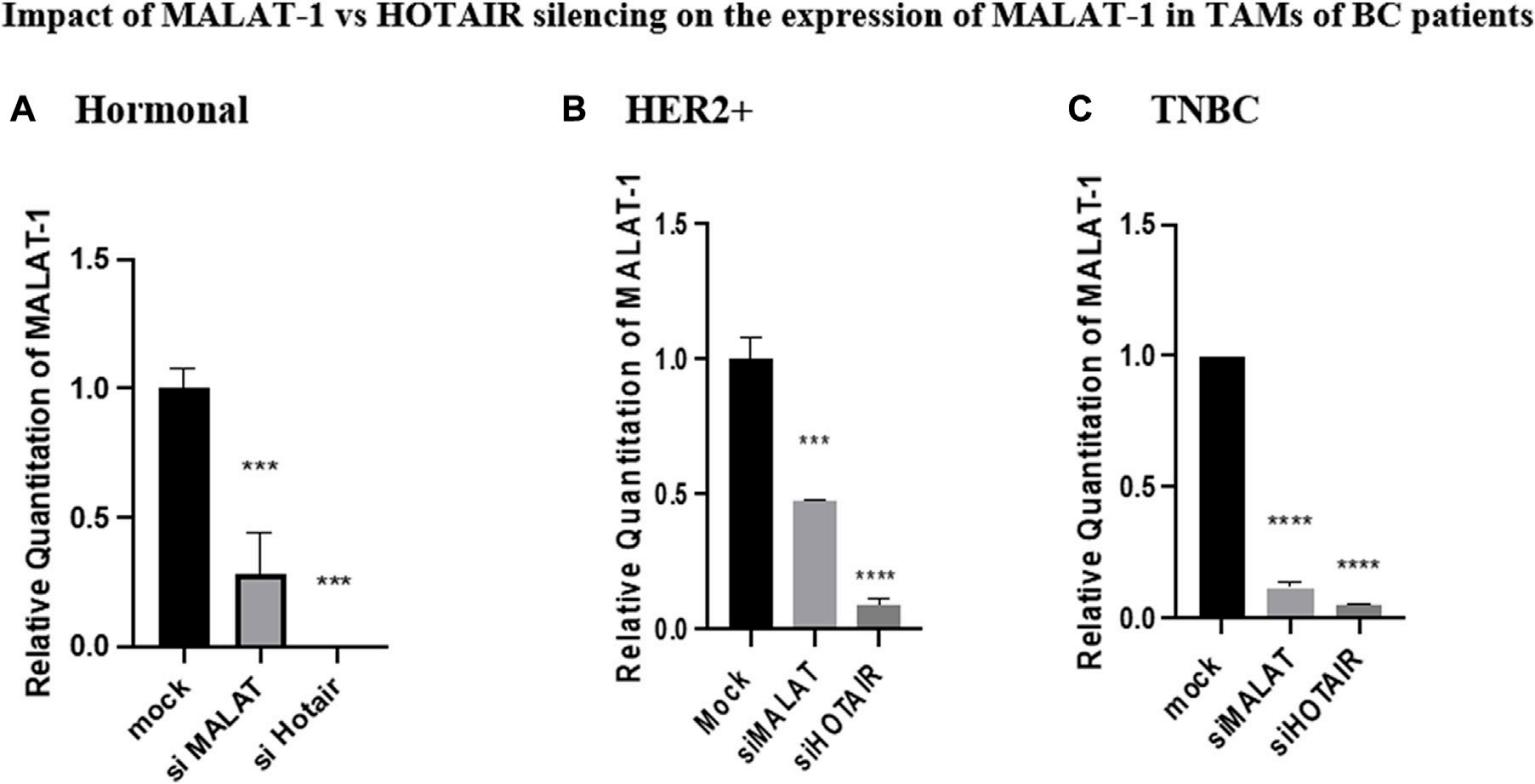
Impact of MALAT1 vs. HOTAIR silencing on the expression of MALAT1 in TAMs of **(A)** hormonal, **(B)** HER2+, and **(C)** TNBC. MALAT1 mRNA levels were measured using qRT-PCR and were normalized to β-actin (endogenous control). The results showed that HOTAIR and MALAT1 silencing resulted in a statistically significant downregulation in MALAT1 expression in the hormonal, HER2+, and TNBC subtypes when compared to the untransfected controls (mocks) of the three subtypes with more significant downregulation of MALAT1 upon silencing of HOTAIR than that observed upon silencing of MALAT1 itself. One-way Anova followed by Dunette’s test of multiple comparison was used for the purpose of comparison between more than two different studied groups. **** = *p* < 0.0001, *** = *p* < 0.001, ** = *p* < 0.01, * = *p* < 0.05, and ns = statistically not significant.

Silencing of HOTAIR in TAMs of different BC subtypes resulted in a decrease in MALAT1 expression in the hormonal, HER2+, and TNBC subgroups (*p* = 0.0009, <0.0001, and <0.0001, respectively) in comparison to untransfected controls (mocks). Unexpectedly, in the three subtypes, MALAT1 showed a more significant downregulation upon silencing with HOTAIR than upon silencing with MALAT1 itself in hormonal, HER2+, and TNBC (*p* = 0.0003, 0.0002, and <0.0001, respectively) in comparison to untransfected TAMs (mocks), as shown in Figure 4.

### Impact of MALAT1 silencing on the expression of HOTAIR in TAMs of BC patients

**FIGURE 5:**
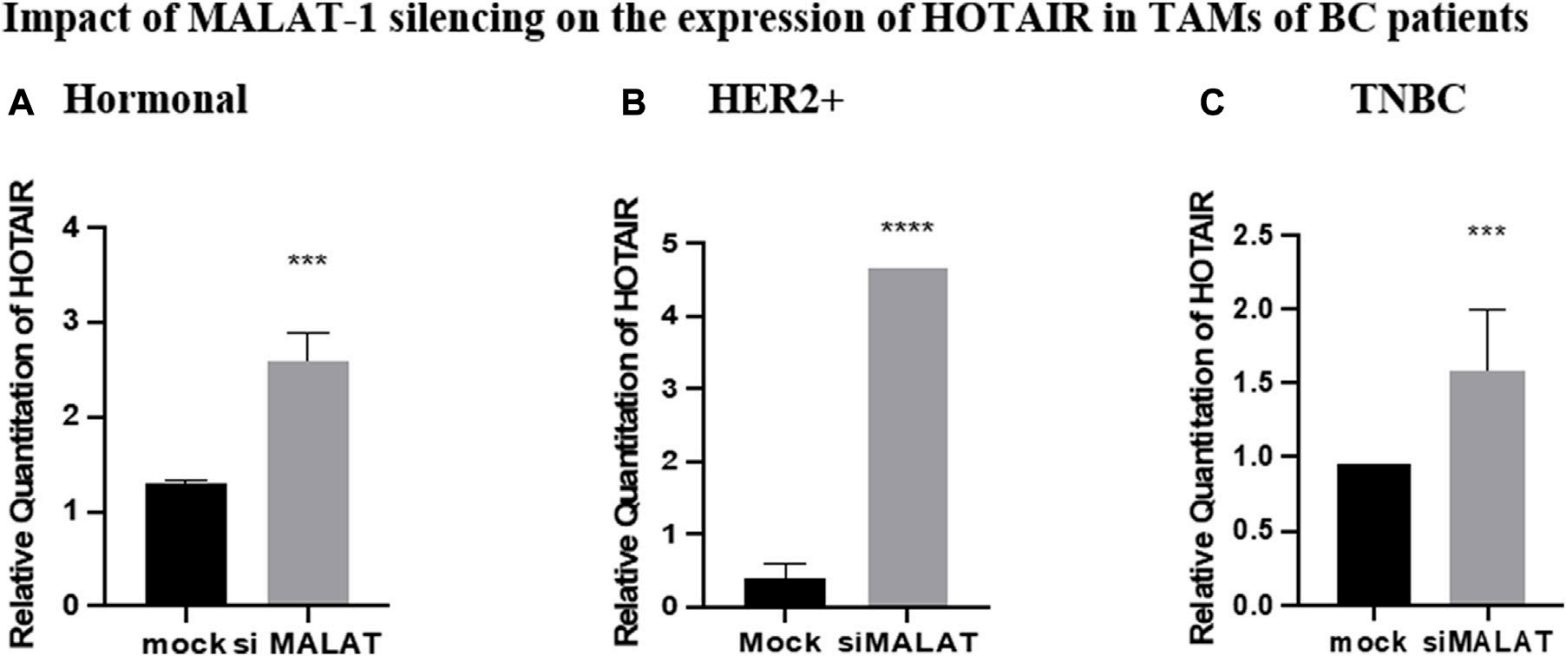
Impact of MALAT1 silencing on the expression of HOTAIR in TAMs of **(A)** hormonal, **(B)** HER2+, and **(C)** TNBC. HOTAIR mRNA levels were measured using qRT-PCR and were normalized to β-actin (endogenous control). The results showed that MALAT1 silencing resulted in statistically significant upregulation in HOTAIR in TAMs of the three subtypes when compared to the untransfected controls (mocks). Parametric student *t*-test was used for comparing two groups **** = *p* < 0.0001, *** = *p* < 0.001, ** = *p* < 0.01, * = *p* < 0.05, and ns = statistically not significant.

Silencing of MALAT1 in TAMs of different subtypes resulted in an increase in the expression of HOTAIR in hormonal, HER2+, and TNBC TAMs (*p* = 0.0008, <0.0001, and 0.0009, respectively) in comparison to untransfected controls (mocks). HOTAIR mRNA levels were quantified using qRT-PCR and normalized to β-actin as an endogenous control, as shown in Figure 5.

### Impact of MALAT1 and HOTAIR silencing on the expression of CD80 in TAMs of BC patients

**FIGURE 6:**
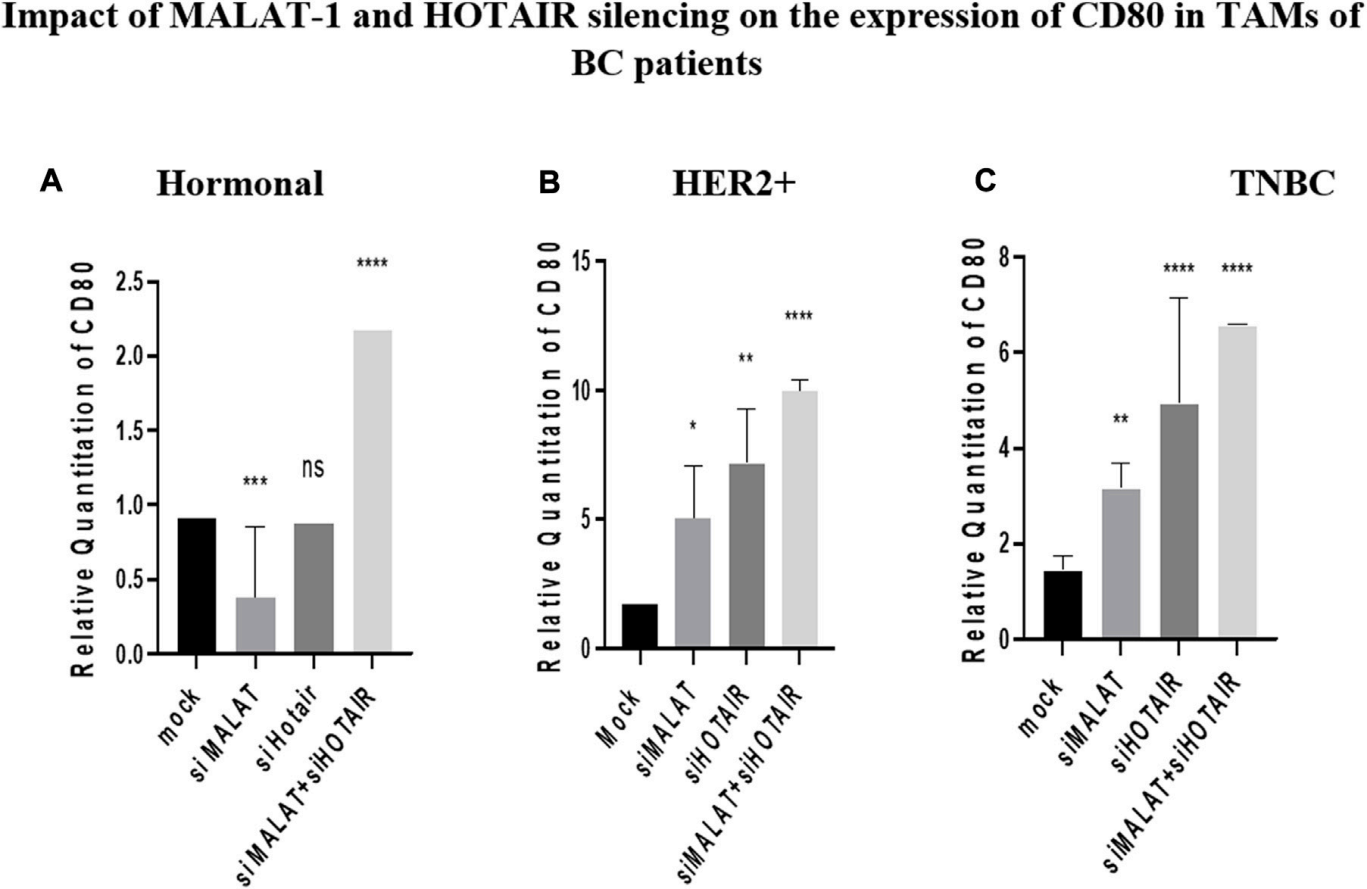
Impact of MALAT1 and HOTAIR silencing on the expression of CD80 in TAMs of **(A)** hormonal, **(B)** HER2+, and **(C)** TNBC. CD80 mRNA levels were measured using qRT-PCR and were normalized to β-actin (endogenous control). The results show that MALAT1 silencing resulted in statistically significant upregulation in CD80 in HER2+ and TNBC subtypes; however, more significant upregulation of CD80 was observed on transfection with silencer against HOTAIR in comparison to untransfected controls (mocks). Conversely, CD80 expression was downregulated upon silencing MALAT1 in TAMs of the hormonal subtype while showing non-significant expression upon silencing of HOTAIR in comparison to untransfected controls (mocks). Meanwhile, co-transfection of HOTAIR silencers with MALAT1 silencers resulted in a dramatic upregulation in the three subtypes in comparison to untransfected controls (mocks). One-way Anova followed by Dunette’s test of multiple comparison was used for the purpose of comparison between more than two different studied groups. **** = *p* < 0.0001, *** = *p* < 0.001, ** = *p* < 0.01, * = *p* < 0.05, and ns = statistically not significant.

CD80 was found to be inversely correlated with MALAT1 in HER2+ and TNBC TAMs, its expression was upregulated by siMALAT1 (*p* = 0.0478 and 0.0012 respectively), while the upregulation was more remarkable by siHOTAIR in HER2+ and TNBC TAMs (*p* = 0.0013 and <0.0001, respectively) in comparison to untransfected controls (mocks). Conversely, in hormonal BC TAMs, CD80 was downregulated by siMALAT1 (*p* = 0.0007) in comparison to untransfected controls (mocks). MALAT1/HOTAIR co-silencing was able to upregulate CD80 in TAMs of hormonal, HER2+ and TNBC (*p*= <0.0001) in comparison to untransfected controls (mocks), as shown in Figure 6.

### Impact of MALAT1 and HOTAIR silencing on the expression of MSLN in BC patients

MSLN was found to have a similar pattern of expression as CD80, where it was inversely correlated with MALAT1 and HOTAIR in HER2+ and TNBC TAMs. Its expression was upregulated by siMALAT1 (*p* = 0.0367 and 0.0015), while the upregulation was much remarkable by siHOTAIR in HER2+ and TNBC TAMs (*p* = 0.0066 and 0.0003, respectively) in comparison to untransfected controls (mocks). However, MSLN expression showed no significant change in the expression in TAMs of the hormonal subtype upon silencing of HOTAIR or MALAT1 in comparison to untransfected controls (mocks). Meanwhile the most significant upregulation for MSLN was found upon MALAT1/HOTAIR co-silencing in hormonal, HER2+, and TNBC (*p*= <0.0001, 0.0002, and <0.0001, respectively) in comparison to untransfected controls (mocks).

### Impact of MALAT1 and HOTAIR knockdown on VEGF-A protein release from TAMs of BC

As a functional analysis, the ELISA technique was used to measure the VEGF-A in the TAMs of the three subgroups (hormonal, HER2+, and TNBC) on silencing MALAT1 or HOTAIR or co-silencing both lncRNAs together in comparison to untransfected TAMs (mocks) corresponding to each subtype. It was shown that VEGF-A was downregulated upon silencing MALAT1 and HOTAIR and co-silencing MALAT1 and HOTAIR in the hormonal BC subtype (*p* = 0.0155, 0.0330, and 0.0199) compared to mock cells. The same downregulation expression profile was observed in the HER2+ subtype upon silencing MALAT1 and HOTAIR and co-silencing MALAT1 and HOTAIR (*p* = 0.0006, 0.0015, and 0.0002) compared to mock cells. Finally, TAMs of TNBC also showed a significant downregulation upon silencing MALAT1 and HOTAIR and co-silencing both of them together (*p* = 0.0009, 0.0025, and 0.0007) compared to untransfected controls (mocks).

### Impact of HOTAIR/MALAT1 knockdown on the cytotoxicity of PDL-1 inhibitor-treated TNBC CD8^+^ cells

LDH toxicity assay was performed to evaluate the cytotoxicity potential of CD8^+^ T cells upon co-culturing with MDA-MB-231 cell line with and without the addition of PDL-1 inhibitor drug. CD8^+^ T cells were cultured under three conditions of treated TAMs-conditioned media (siMALAT1, siHOTAIR, and siMALAT1+siHOTAIR). The results showed that the cytotoxicity percentage of CD8^+^ cells upon culturing in siMALAT1, siHOTAIR, and siMALAT1/siHOTAIR TAMs had an average of approximately 76.6% cytotoxicity; however, upon the addition of the PDL-1 inhibitor on CD8^+^ T cells that is previously cultured in siMALAT1, siHOTAIR, and siMALAT1/siHOTAIR TAMs-conditioned media, the cytotoxicity percentages had an average of approximately 61.3% and upon addition of the PDL-1 inhibitor alone on CD8+/MDA-MB-231 co-culture, the cytotoxicity was 79%.

### Efficiency of CD14^+^ monocyte differentiation to TAMs

The morphological change was assessed to confirm the differentiation efficiency of CD14^+^ monocytes to TAMs. CD14^+^ monocytes were isolated from total PBMCs by negative selection using magnetic nanobeads against antibodies of all cells other than CD14^+^ monocytes. Freshly isolated CD14^+^ monocytes were observed under the microscope. Examination showed small, spherical-shaped cells with a smooth surface. However, upon culturing CD14^+^ monocytes for 7 days in culture media supplemented with anti-inflammatory ILs (IL-10, IL-4, and M-CSF) with the addition of TCM, morphological change was observed where the cells became larger with a non-smooth edgy surface.

### CD8^+^ T-cell isolation purity

To assess the isolation purity of CD8^+^ T cells, the flow cytometry technique was conducted using anti-CD8^+^ PE after isolating the CD8^+^ T cells from PBMCS using magnetic nanobeads. The results showed that the isolation purity reached 70.2%.

## Discussion

Breast cancer (BC) is one of the most commonly diagnosed cancers in women worldwide, accounting for 11.7% of all cancer cases in 2020. It is classified molecularly according to the expression of estrogen receptor (ER), progesterone receptor (PR), and human epidermal growth factor receptor 2 (HER2) with the percentage of the proliferating index (Ki67). Accordingly, BC is classified into five major subclasses, which are luminal A, luminal B HER2-, luminal B HER2+, HER2 enriched, and TNBC. Luminal A is the most abundant subtype having a better prognosis than luminal B. Furthermore, HER2+, which is a hormonal negative subtype, has a worse prognosis than the hormonal subtypes despite the presence of anti-HER2-targeted therapy “for example, trastuzumab.” Finally, TNBC (basal-like) has the worst and most aggressive prognostic profile of all the subtypes (Kondov et al., 2018).

The most common BC treatment strategies are hormonal therapy, anti-HER2-targeted therapy, and chemotherapy. Both hormonal and anti-HER2-targeted therapies are limited to specific subtypes of BC. Moreover, chemotherapy has the ability to target all BC subtypes but causes profoundly aggressive side effects (Yerushalmi et al., 2009). In this context, targeted immunotherapy can be a new therapy alternative to overcome the limitations of other therapeutic strategies.

In general, tumors have the ability to recruit stromal cells (e.g., fibroblasts), immune myeloid cells, and vascular cells by the secretion of cytokines building up a tumor microenvironment (TME) by releasing growth-promoting signals and remodeling the tissue structure affecting initiation, progression, metastasis, vascularization, and therapy responses. During the last decades, cancer treatment strategies focus only on the cancer cell ignoring the TME, which is a key player in the tumor progression (Allaoui et al., 2016). Within the TME, CD14^+^ monocytes can infiltrate and differentiate into tumor-associated macrophages (TAMs) that can reach 40% of the tumor’s volume. TAMs are known to have pro-inflammatory phenotype (M1) in the early stages of cancer while having anti-inflammatory (M2) phenotype once cancer starts progressing in suppressing many immune cells. The differentiation to M2 takes place in the presence of some anti-inflammatory cytokines in the TME, namely, IL-10, IL-4, IL-13, and M-CSF (Zhou et al., 2020). In addition to TAMs correlation with tumor progression, there are some immunomodulatory proteins that have been reported in the last years to be overexpressed in various types of cancers, especially CD80 and mesothelin (MSLN). Furthermore, in the past few years, the role of non-coding RNAs in the pathogenesis of BC has been of significant importance and their correlation with immunomodulatory proteins, as shown in our previous study (Hussein et al., 2022). Interestingly, HOTAIR and MALAT1 are known to be the master regulators of tumor progression (Aiello et al., 2016).

In our previous work, MALAT1 was shown to be upregulated in BC tissues, especially TNBC epigenetically upregulating MSLN; Barsoum et al. (2020) proposed that there may be a possible interlinkage between MALAT1 and immunomodulatory proteins. The aim of the current study is to investigate if MALAT1 will give the same pattern of expression in TAMs of the hormonal, HER2+, and TNBC, highlighting that its role in cancer is still controversial whether it is an oncogenic or a tumor suppressor lncRNA. Additionally, HOTAIR is another lncRNA that is known to function as an oncogenic lncRNA as previously mentioned. Therefore, the aim of this work is to study the interlinkage between MALAT1 and HOTAIR and their effect on the expression of oncogenic immunomodulatory proteins in TAMs of the hormonal, HER2+, and TNBC subtypes.

To fulfill this aim, MALAT1 and HOTAIR expressions were first screened. MALAT1 was found to be over expressed in hormonal, HER2+, and TNBC compared to controls, as shown in Figure 1A. This finding aligns with our previous studies confirming that MALAT1 is overexpressed in BC tissues more than healthy tissues (Barsoum et al., 2020). Nevertheless, another research study observed the overexpression of MALAT1 in many BC cell lines (MCF-7, SK-BR-3, MDA-MB-468, MDA-MB-231, T-47d, and MDA-MB-453) compared to normal breast epithelial cell line (MCF-10A) (Yue et al., 2020). Moreover, MALAT1 was shown to be overexpressed in other cancers such as multiple myeloma (Liu et al., 2020) and hepatocellular carcinoma (HCC) (Lai et al., 2012).

The screening of HOTAIR expression in TAMs of hormonal, HER2+, and TNBC also showed a significant upregulation in BC samples compared to healthy samples, as shown in Figure 1B. This finding is supported by another study that was conducted on MCF-7 and MDA-MB-231, showing significant upregulation of lncRNA HOTAIR when compared to MCF-10A (Wang et al., 2020). Moreover, HOTAIR is shown to be not only overexpressed in BC but also in ovarian cancer (Qiu et al., 2014); thus, suggesting that HOTAIR and MALAT1 are oncogenic lncRNAs.

Therefore, it was tempting to study the impact of lowering the expression of MALAT1 and HOTAIR in TAMs. Our research group has previously studied the role of these lncRNAs along with their candidate downstream targets, immunomodulatory proteins, CD80, and MSLN in TNBC tissues and MDA-MB-231 (Barsoum et al., 2020). However, here, this study is concerned with their expression pattern in TAMs.

CD80 and MSLN were found to be overexpressed in HER2+ and TNBC while showing a non-significant change of expression on examining the hormonal subtype, as shown in Figure, suggesting that there is another key player regulating the expression of CD80 in the hormonal subtype and preventing its overexpression. This key player may be estrogen that is known in the literature to downregulate CD80 levels. It was previously observed that estrogen has an inverse correlation with CD80, as shown in a study conducted on RAW264.7 cell line (Yang et al., 2016). Highlighting the fact that RAW264.7 is a murine macrophages cell line, similarity in the expression is expected. Moreover, another research group showed that estrogen promotes the B cell activity *in vitro* by downregulating CD80 expression (Fu et al., 2011). Thus, concluding that estrogen may be the factor inhibiting CD80 to upregulate.

Upon screening of MSLN in TAMs of the three subgroups, it was found that MSLN is also overexpressed in HER2+ and TNBC, while it was significantly downregulated in the hormonal subtype, as shown in Figure 2B, suggesting that estrogen plays a role in this downregulation. This finding is partially supported by a study that screened 99 primary BC samples by immunohistochemistry analysis using formalin-fixed paraffin-embedded archival tumor tissues confirming that MSLN was only overexpressed in TNBC (67%) in contrast to its rare expression in the hormonal and the HER2+ subtypes (Tchou et al., 2012). These data partially support our findings that MSLN is not upregulated in the hormonal subtype (possibly due to the presence of estrogen), however, contradicting with our data that suggest MSLN to be upregulated in HER2+. In another study, MSLN expression was detected in 77 cases out of 482 patients (16.0%) and was the highest in TNBC (31/75; 41.3%), followed by the HER2+ subtype (6/33, 18.2%), and then the luminal subtype (36/374; 9.6%) (Suzuki et al., 2020). Moreover and more interestingly, MSLN was shown in our previous work to be upregulated in BC subtypes, especially TNBC (Barsoum et al., 2020).

To observe the impact of knocking down of MALAT1 and HOTAIR in TAMs of the three BC subtypes (hormonal, HER2+, and TNBC), the correlation between MALAT1 and HOTAIR was first observed showing that on the silencing of HOTAIR, MALAT1 was downregulated in all subtypes of BC, as shown in Figure 4, suggesting that HOTAIR may be an upstream regulator for MALAT1.

Moreover, upon silencing MALAT1, HOTAIR expression was upregulated, and this was observed in the three subtypes, as shown in Figure 5, suggesting that HOTAIR expression may have increased as a compensatory mechanism for the loss of MALAT1 expression. These findings suggest that MALAT1 expression is directly correlated with HOTAIR expression; however, it was reported that HOTAIR and MALAT1 have opposite expression profiles in estrogen-mediated transcriptional regulation in prostate cancer cells (Rinn et al., 2007). These differences may be due to cancer specificity or tissue specificity as this study observed cancer cell lines in prostate cancer; however, our study is on TAMs in BC. Moreover, the opposite expression profile may be due to the fact that estrogen has an opposite correlation with MALAT1 expression in prostate cancer, as shown in the mentioned study, thus upon downregulation of MALAT1, HOTAIR is overexpressed as a compensatory mechanism, thus having an inverse correlation. Knowing that in the same study upon treatment of the BC cell line with estrogen, no change in the expression of MALAT1 was observed (Aiello et al., 2016), thus no correlation between estrogen and MALAT1 expression in BC supporting our identical finding that MALAT1 and HOTAIR show same overexpression patterns in the three subgroups regardless the hormonal expression. Furthermore, in another study that aligns with our finding states that MALAT1 and HOTAIR have a positive correlation with each other after conducting a correlation analysis between their serum levels in BC (El-Fattah et al., 2021). Unexpectedly, upon comparing MALAT1 expression after silencing its gene *vs.* silencing HOTAIR, it was shown that the HOTAIR knockdown effect was more significant in downregulation of MALAT1 compared to knocking down of MALAT1 itself, as shown in Figure 4.

As a consequence of the CD80 overexpression in HER2+ and TNBC TAMs, thus highlighting the possibility of being an oncogenic immunomodulatory protein (Li et al., 2020), it was worth observing the effect of MALAT1 as an important lncRNA in the regulation of CD80. Unexpectedly, our data showed that upon silencing of MALAT1, CD80 expression was upregulated, thus having an inverse correlation in HER2+ and TNBC, as shown in Figures 6B,C, respectively. This finding aligns with another study showing MALAT1 and CD80 to be inversely correlated in A549 cells (neonatal respiratory distress syndrome) (Juan et al., 2018). MALAT1 and CD80 inverse correlation was also supported in another research study that suppressed MALAT1 in the dendritic cells (Wu et al., 2018). Observing this inverse correlation in dendritic cells validate our data since that macrophage (TAMs) and dendritic cells are both derived from the same progenitor (monocytes), thus having similarities in the expression can be expected.

CD80 is a ligand for two receptors on T cells, namely, CD28 and CTLA4. T cells are activated upon binding of CD80 and CD28, thus having a pro-inflammatory activity. However, on binding with CTLA4, T cells were found to be suppressed causing energy and anti-inflammatory activity. Surprisingly, it was found that CTLA4 has a higher affinity to bind with CD80 than CD28 (Lai et al., 2012). Taking this into account, this study proposes that the overexpression pattern of CD80 in TAMs of the three subgroups, as shown in Figure 2, occurs due to the immune-suppressive effect of CD80 due to its binding to CTLA4 on T cells. However, CD80 shifts its binding toward CD28 upon silencing MALAT1, thus its overexpression has immunostimulatory activity, and this may happen due to the downregulation of CTLA4 as a result of downregulation of MALAT1. The relationship between MALAT1 and CTLA4 is not previously studied in cancer; however, its data in asthma showed that MALAT1 sponges miR-155 upregulating CTLA4 (Liang and Tang, 2020). In other words, our study proposes that the overexpression of MALAT1 in TAMs might have upregulated CTLA4, thus upregulating CD80 to enhance its immunosuppressive activity and build up an environment favorable for its tumorigenic role. Furthermore, it was expected that CD80 to be downregulated upon the silencing of MALAT1 but unexpectedly an inverse correlation was observed. In this context, we propose that this might have happened after silencing MALAT1, thus consequently and considerably impacting the oncogenic potential of TAMs making the CD80 regain its potential (in the absence of MALAT1) to function as a pro-inflammatory protein, thus upregulation was observed.

Focusing onto the hormonal subtype, CD80 expression was observed to be significantly downregulated upon silencing MALAT1, as shown in Figure 6A. This could be due to the dominancy of the effect of estrogen in downregulating CD80. Estrogen might have the potential to abolish the upregulation effect caused by MALAT1 silencing. This finding supports our previous conclusion that estrogen and CD80 are inversely correlated to each other, and upon analyzing the effect of silencing of HOTAIR on the expression profile of CD80, the same expression pattern was observed, in which CD80 was upregulated in HER2+ and TNBC, as shown in Figures 6B,C, respectively. The correlation between CD80 and HOTAIR is not studied before, thus in this context, this is considered the first research study to be conducted focusing on HOTAIR and CD80 correlation.

Focusing on to the hormonal subtype, CD80 expression is shown to have a non-significant change in the expression upon HOTAIR silencing compared to mock, as shown in Figure 6A.

MSLN is known to be an oncogenic immunomodulatory protein in many types of cancers, for example ovarian, adenocarcinoma, and most importantly BC (Wang et al., 2020). Our previous work confirmed that MSLN is overexpressed in BC tissues and cell lines, thus functioning as an oncogenic protein (Barsoum et al., 2020). So, it was a good candidate to examine its correlation with MALAT1 and HOTAIR.

**FIGURE 7:**
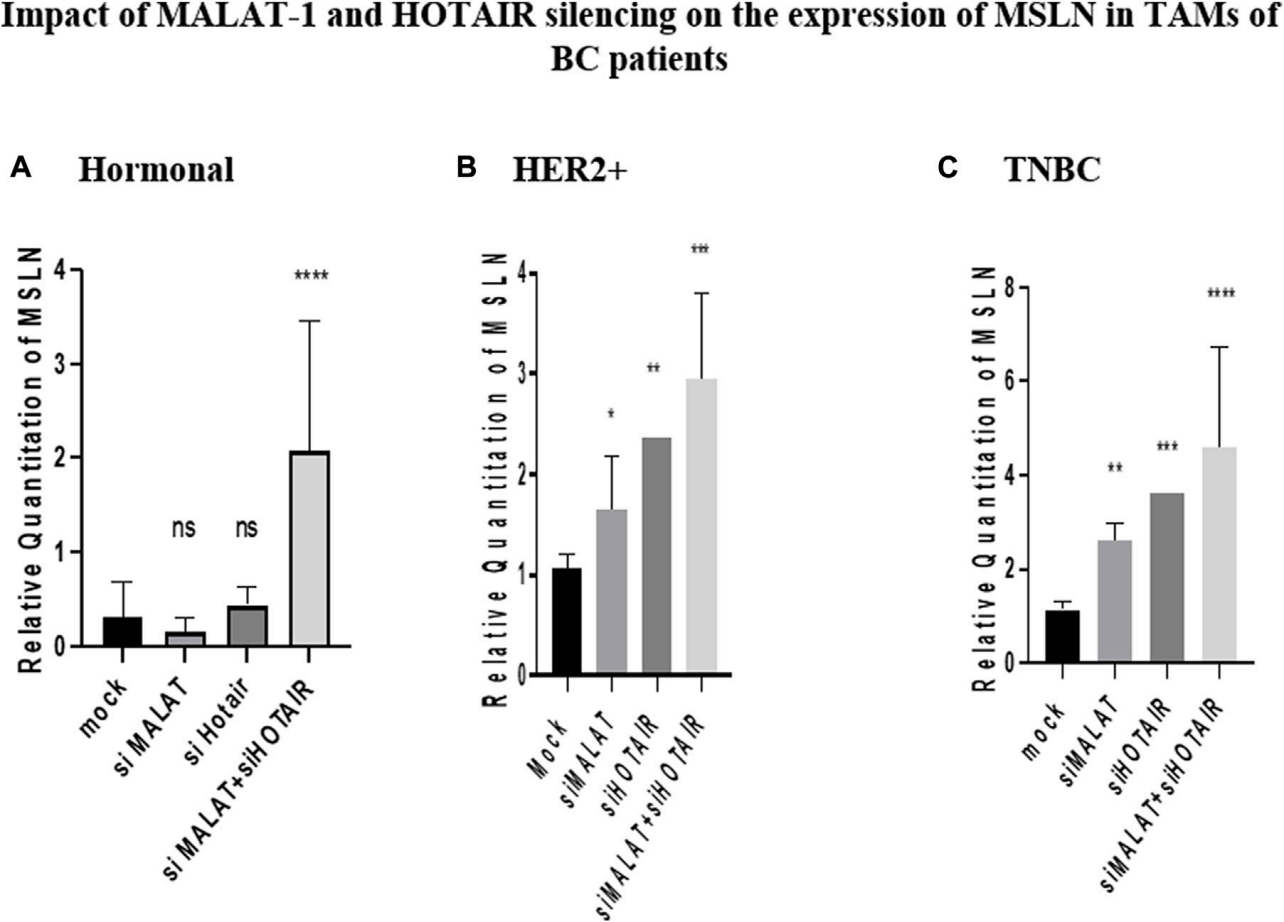
Impact of MALAT1 and HOTAIR silencing on the expression of MSLN in TAMs of **(A)** hormonal, **(B)** HER2+, and **(C)** TNBC. MSLN mRNA levels were measured using qRT-PCR and were normalized to β-actin that functioned as an endogenous control. The results show that MALAT1 silencing resulted in statistically significant upregulation of MSLN in HER2+ and TNBC subtypes; however, more significant upregulation of MSLN was observed on transfection with silencer against HOTAIR when compared to the untransfected controls (mocks) of the same subtypes. Conversely, MSLN expression showed no significant change in expression upon silencing MALAT1 or HOTAIR in TAMs of the hormonal subtype compared to the untransfected controls (mocks) of the same subtype. Meanwhile, the co-transfection of HOTAIR silencers with MALAT1 silencers resulted in the most significant upregulation profile for MSLN in TAMs of the three subgroups in comparison to untransfected controls (mocks). One-way Anova followed by Dunette’s test of multiple comparison was used for the purpose of comparison between more than two different studied groups. **** = *p* < 0.0001, *** = *p* < 0.001, ** = *p* < 0.01, * = *p* < 0.05, and ns = statistically not significant.

Upon silencing MALAT1, MSLN expression had the same pattern as CD80 in both HER2+ and TNBC. Unexpectedly, upon silencing MALAT1, MSLN expression was upregulated in both non-hormonal subtypes: HER2+ and TNBC, as shown in Figures 7B,C, respectively. The relationship between MALAT1 and MSLN is not studied except in our previous work on TNBC tissues that showed downregulation of MSLN upon MALAT1 silencing (Barsoum et al., 2020). The contradiction might be due to the difference in the cell type studied as our previous work was on the cancer cell; however, this work focused on TAMs (immune cell).

It is important to highlight that MSLN overexpression correlates to the increased levels of soluble MSLN that by its turn binds to CD206 (mannose receptor) *via* GPI anchor facilitating macrophages polarization to TAMs (Dangaj et al., 2011). In this context and as mentioned before it was unexpected to observe an upregulation in MSLN expression after MALAT1 silencing. MALAT1 silencing consequently and considerably impacted the oncogenic potential of TAMs. The unexpected upregulation of MSLN upon MALAT1 silencing might have happened as a compensatory mechanism performed by TAMs for the loss of MALAT1. Upon upregulation of MSLN, soluble MSLN levels would also increase, thus increasing the binding potential to CD206. As a consequence, the polarization of macrophages to TAMs would be enhanced.

Focusing on to the hormonal subtype, MSLN gives a close expression pattern to CD80, its change in expression is non-significant compared to controls upon MALAT1 silencing, as shown in Figure 7A, proposing again that estrogen is a factor playing an important role in downregulating MSLN, thus balancing the upregulation effect caused by MALAT1 silencing in the hormonal subtype leading to a non-significant change in expression of MSLN compared to mocks.

Upon silencing HOTAIR, MSLN gave the same pattern of expression on silencing with MALAT1 in HER2+ and TNBC. MSLN was shown to be upregulated in both HER2+ and TNBC, as shown in Figures 7B,C, respectively. Interestingly, the correlation between HOTAIR and MSLN is not been previously studied on any cell type, thus studying this correlation in TAMs highlights the importance of investigating this relationship in other cell types either cancer cells or immune cells.

On comparing the MSLN or CD80 expression upon silencing with HOTAIR and upon silencing with MALAT1 in both HER2+ and TNBC, immunomodulatory proteins are found to be upregulated more significantly upon silencing with HOTAIR, as shown in Figures 6B,C and Figures 7B,C. This is may again be due to that HOTAIR is the upstream regulator for MALAT1 as previously mentioned having a greater effect on the downregulation of MALAT1 than knockdown of MALAT1 itself. Thus, upon silencing HOTAIR, MALAT1 is abolished, making the downregulation of MALAT1 significantly remarkable and consequently more significant upregulation for immunomodulatory proteins was observed.

Co-transfection of silencers against MALAT1 with silencers against HOTAIR was expected to affect the expression of CD80 and MSLN more significantly than silencing each lncRNA on its own. Thus, upon silencing both lncRNAs, CD80 and MSLN were upregulated more significantly compared to MALAT1 or HOTAIR separately in HER2+ and TNBC, as shown in Figures 6B,C and Figures 7B,C.

As for the hormonal subtype, a significant upregulation of CD80 and MSLN was observed upon co-transfection of silencers against MALAT1 and HOTAIR (Figure 6A and Figure 7A). These results were expected due to the dual effect of the simultaneous silencing of HOTAIR and MALAT1 and their success to dominate the effect of downregulation caused by estrogen, confirming that, in general, MALAT1 and HOTAIR are inversely correlated with CD80 and MSLN in the three subtypes, but estrogen can dominate in the hormonal subtype causing the effect of silencing HOTAIR and MALAT1 to be masked.

As previously mentioned, there are various studies that tackled the role of MALAT1 and HOTAIR functionally as oncogenic lncRNAs in breast cancer tissues. However, TAMs were still questionable and not confirmed whether they were functioning as oncogenic or tumor suppressor lncRNAs. For this reason, one of the functional analyses for oncogenesis and metastasis was observed to be a confirmatory tool for the role of these two lncRNAs. Vascular endothelial growth factor A (VEGF-A) was chosen as a suitable candidate for the functional analysis for two major reasons: first, to confirm that TAMs have a role in metastasis through VEGF-A, highlighting that the role of TAMs in metastasis is functionally analyzed for the first time, and the second reason was that VEGF-A is considered a reflection for the metastasis thus tumorigenesis, and this will confirm whether MALAT1 and HOTAIR are oncogenic or tumor suppressor.

**FIGURE 8:**
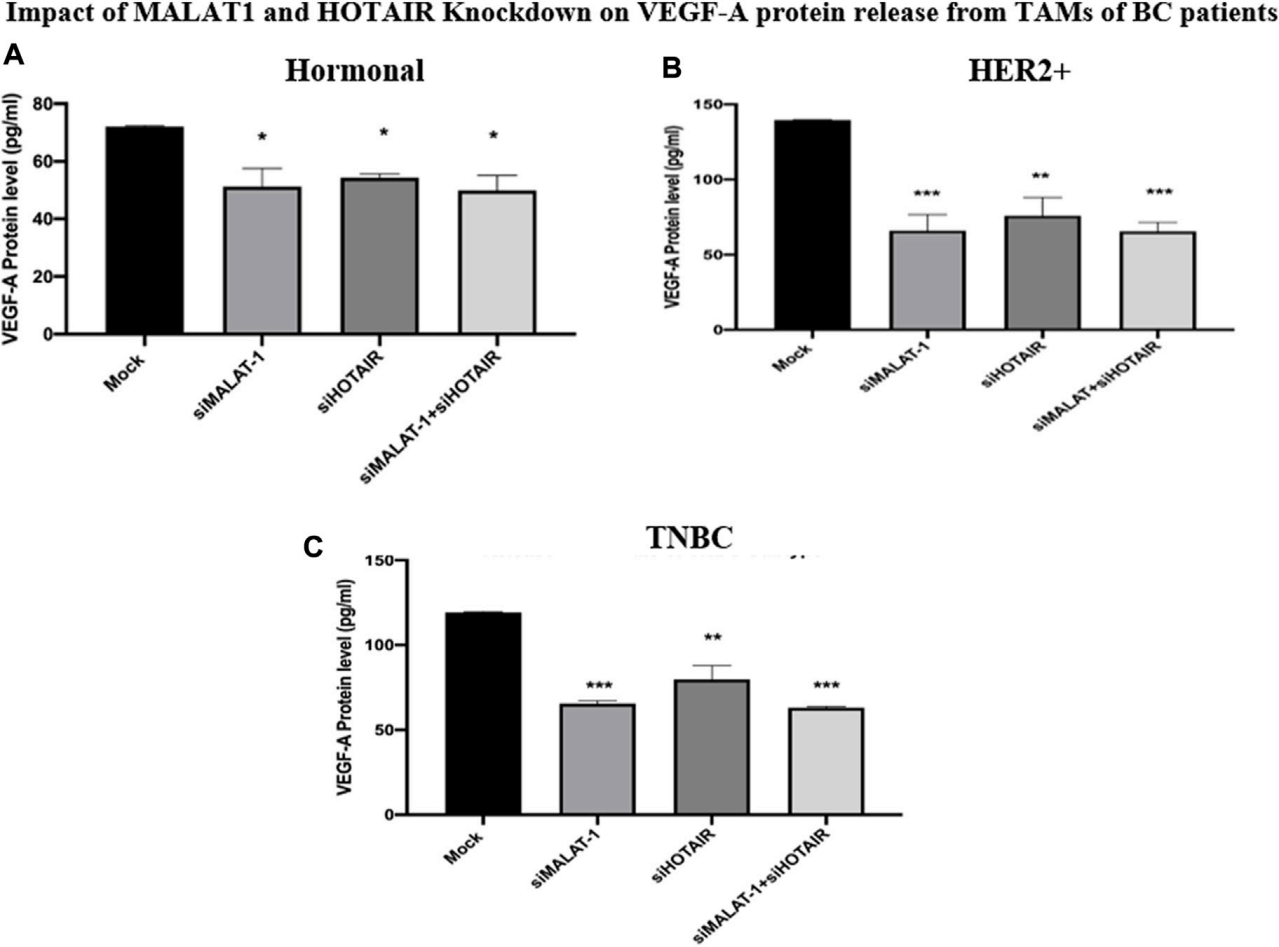
Impact of MALAT1 and HOTAIR knockdown on VEGF-A protein release from TAMs of **(A)** hormonal **(B)** HER2+, and **(C)** TNBC. The ELISA technique was used to measure the levels of VEGF-A in TAMs of the three subtypes. The results showed significant downregulation of VEGF-A protein release from TAMs of the hormonal, HER2+, and TNBC either upon silencing MALAT1 and HOTAIR separately or their co-silencing together in comparison to untransfected controls (mocks). One-way Anova followed by Dunette’s test of multiple comparison was used for the purpose of comparison between more than two different studied groups. **** = *p* < 0.0001, *** = *p* < 0.001, ** = *p* < 0.01, * = *p* < 0.05, and ns = statistically not significant.

The VEGF-A protein level was shown to be downregulated upon silencing lncRNAs whether separately or simultaneously in the hormonal, HER2+, and TNBC, as shown in Figure 8, confirming the idea that MALAT1 and HOTAIR are oncogenic lncRNAs in TAMs.

**FIGURE 9:**
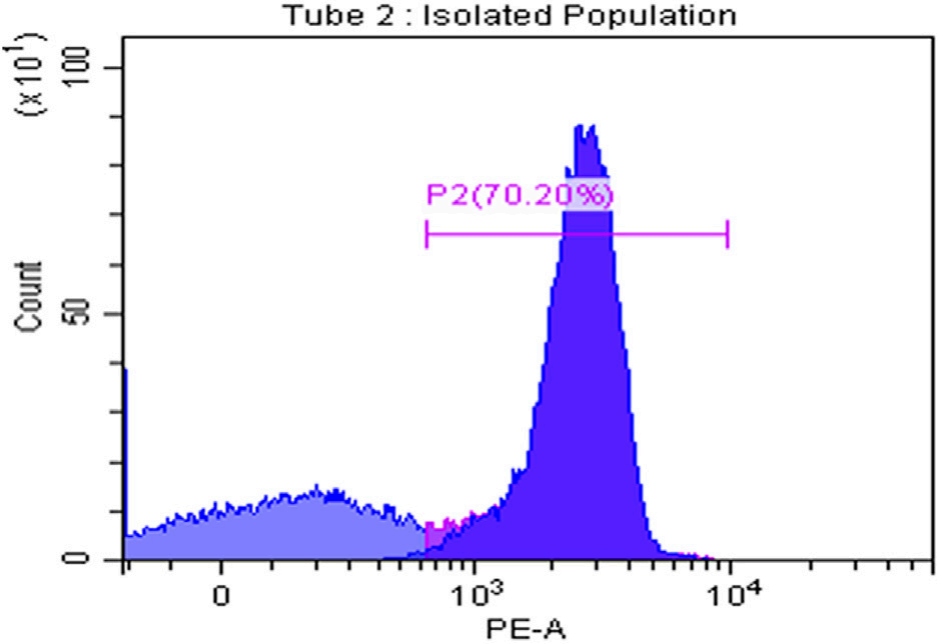
CD8^+^ T cells isolation purity. CD8^+^ T-cell isolation purity was assessed using flow cytometry measuring CD8 self-marker for T cells showing that the isolation was of high purity reaching 70.20%.

**FIGURE 10:**
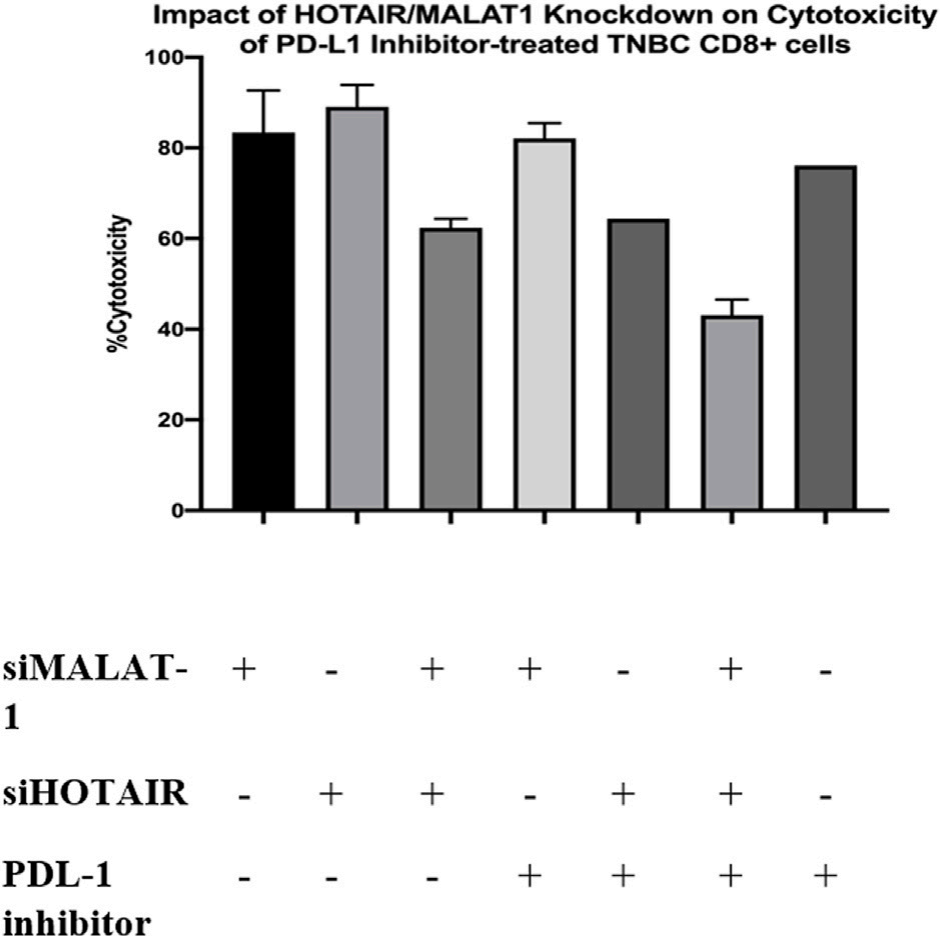
Impact of HOTAIR/MALAT1 knockdown on cytotoxicity of PDL-1 inhibitor-treated TNBC CD8^+^ cells. LDH toxicity assay was performed to assess the cytotoxicity potential of CD8^+^ T cells after culturing in siMALAT1, siHOTAIR, and siMALAT1/siHOTAIR treated TAMs-conditioned media with and without the addition of the PDL-1 inhibitor showing that the cytotoxicity was of higher values upon silencing lncRNAs alone without the addition of PDL-1 inhibitor.

The regulatory role of TAMs on CD8^+^ T cells was for the first time assessed in the presence of MDA-MB-231 cell line. The purity of CD8^+^ T cells isolation was assessed using flow cytometry as shown in Figure 9. CD8^+^ T cells (previously cultured in transfected TAMs-conditioned media) were co-cultured with MDA-MB-231 cell line. As previously mentioned, TAMs have an inhibitory role on CD8^+^ T cells, thus our work aims to observe if the silencing of MALAT1 and HOTAIR will impact this immune-suppressive activity. The immune-suppressive activity was assessed by observing the cytotoxic activity of CD8^+^ T cells on MDA-MB-231 cell line. LDH assay was conducted showing that upon culturing of CD8^+^ T cells in TAMs-conditioned media previously treated with silencers against MALAT1 and HOTAIR, the cytotoxicity activity of CD8^+^ T cells is increased, as shown in Figure 10, supporting the fact that MALAT1 and HOTAIR are oncogenic lncRNAs, and upon their knocking down, the T cells restored its immunostimulant cytotoxic activity.

**FIGURE 11:**
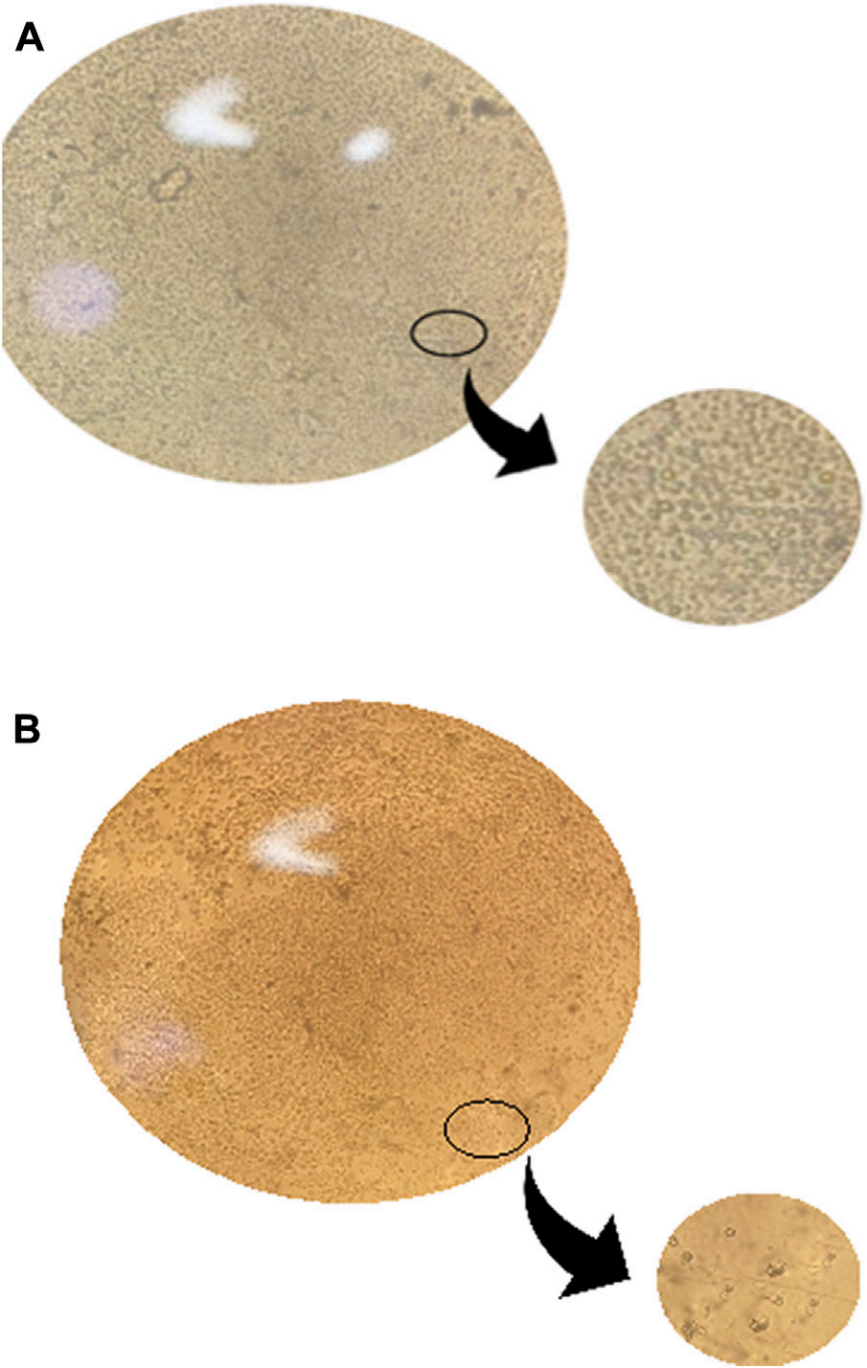
Morphology of CD14^+^ monocytes and TAMs of BC using a microscope. **(A)** Freshly isolated CD14^+^ monocyte’s morphology was examined using a microscope before differentiation. Examination showed cells with a spherical and smooth surface. The cells are considered small in size. **(B)** TAMs morphology was examined using the microscope on the 7th day after differentiation is completed, and examination showed cells morphology changed to be with an edgy and rough surface and a relatively larger size than monocytes.

Programmed cell death protein 1 (PDL-1) is known to be a negative regulator expressed on the surface of T cells. PDL-1 binds to its ligand programmed death ligand-1 (PD-1) on the tumor cell suppressing the activity of CD8^+^ T cell (53). Consequently, upon culturing CD8^+^ T cells with MDA-MB-231 cell line, the cytotoxic activity of T cells is expected to be suppressed, as a result, the PDL-1 inhibitor drug was added to the co-culture media (CD8^+^ T cells cultured in treated TAM-conditioned media) to observe the impact of adding the PDL-1 inhibitor with oncogenic lncRNAs silencing, and whether this addition will cause a synergetic activity increasing the cytotoxicity of CD8^+^. Unexpectedly, the cytotoxicity of CD8^+^ T cells was not increased upon the addition of the PDL-1 inhibitor in comparison to CD8^+^ cytotoxicity upon only silencing of the oncogenic lncRNAs without the addition of PDL-1, as shown in Figure 10. Our study suggests that this might have happened either due to the PDL-1 inhibitor, which does not have any additional role in increasing the cytotoxicity of CD8^+^ T cells or that the dose of PDL-1 inhibitor has to be adjusted knowing that the dose used was 200 nM as indicated in the previous literature using same immune cell (CD8^+^) and same cell line (MDA-MB-231) (Passariello et al., 2019). In all experiments conducted, The efficiency of monocytes differentiation into TAMs was confirmed using microscopic examination as shown in Figure 11.

**FIGURE 12:**
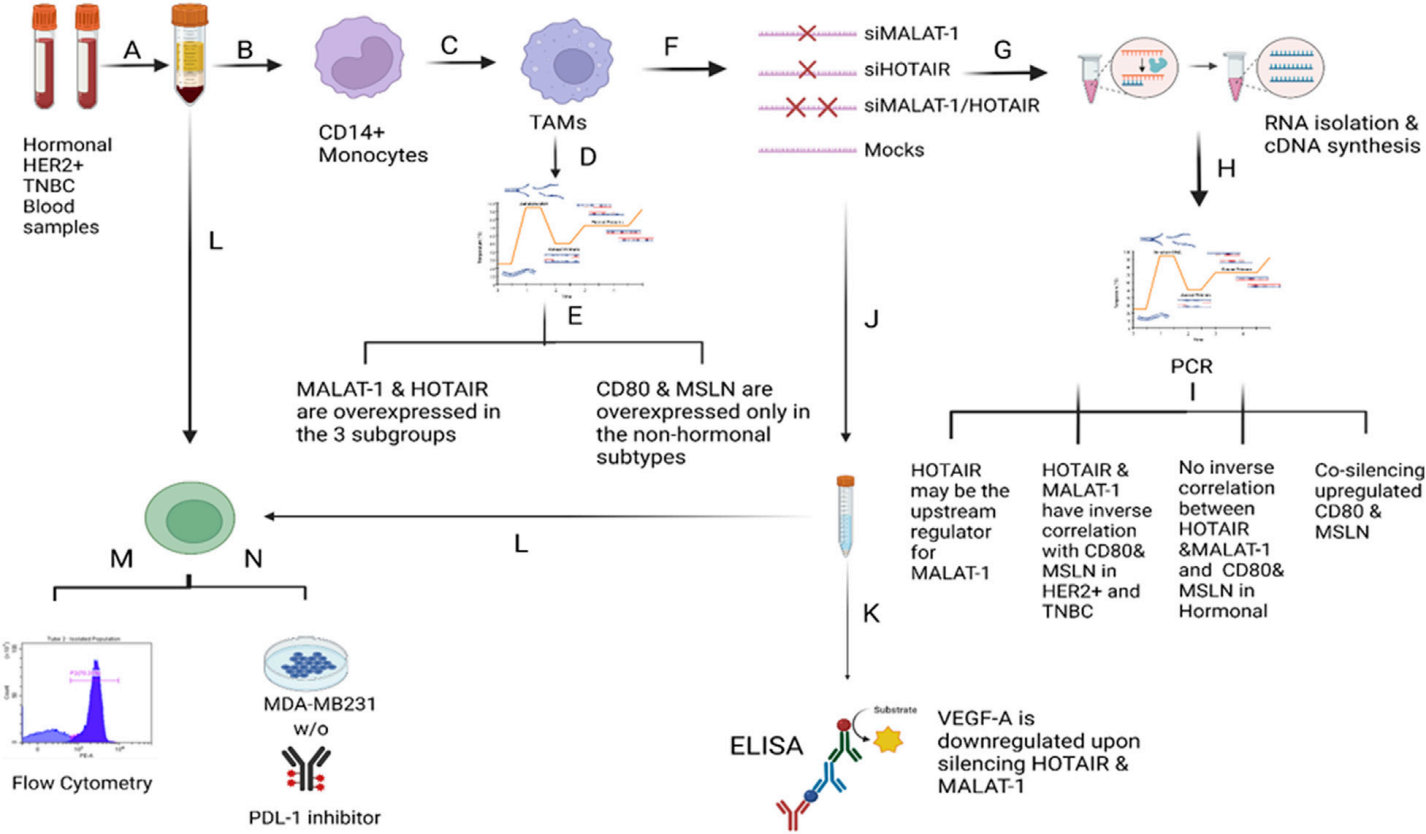
Overview of the methodology and results. A) Total PBMCs were isolated from fresh blood samples collected from BC patients (hormonal, HER2^+^, and TNBC subtypes) using the Ficoll technique. B) CD14^+^ monocytes were isolated from the total PBMCs using the negative depletion method. C) CD14^+^ monocytes were cultured in the differentiation medium (IL-10, IL-4, M-CSF, and TCM) for 7 days. D) On the 7th day, TAMs were harvested and subjected to total RNA isolation, cDNA synthesis and PCR screening for MALAT1, HOTAIR, CD80, and MSLN. E) MALAT1 and HOTAIR were found to be overexpressed in the TAMs of the three subgroups proposing their potential to be oncogenic lncRNAs. CD80 and MSLN were found to be overexpressed only in HER2+ and TNBC TAMs, suggesting that estrogen can be the controlling factor for their expression in the hormonal subtype. F) TAMs were transfected with silencers against MALAT1 and HOTAIR (four conditions of TAMs were prepared: MALAT1 silenced TAMs, HOTAIR silenced TAMs, MALAT1/HOTAIR co-silenced TAMs, and untransfected mocks). G) Total RNA isolation and cDNA synthesis experiments were conducted. I) PCR technique was used to assess the expression of MALAT1, HOTAIR, CD80, and MSLN under four conditions of TAMs concluding that 1) MALAT1 is downregulated upon silencing of HOTAIR in the hormonal and non-hormonal subtypes. 2) CD80 and MSLN are upregulated upon silencing HOTAIR or MALAT1 in non-hormonal TAMs. 3) Estrogen was dominating the effect of the single silencing (of MALAT1 or HOTAIR) in hormonal subtype. 4) Co-silencing (dual silencing of MALAT-1 and HOTAIR) showed an upregulation of the immunomodulatory proteins in the hormonal and non-hormonal TAMs. J) To confirm the oncogenicity of MALAT1 and HOTAIR, the ELISA technique was used to measure VEGF-A protein observing that upon silencing, VEGF-A protein is downregulated in the hormonal and non-hormonal TAMs. L) CD8^+^ T cells were isolated from total PBMCs through negative depletion and were cultured under four conditions of the TAMs-conditioned media. M) Flow cytometry measuring the CD8^+^ marker was used to confirm the purity of isolation. N) LDH toxicity assay was performed to assess the cytotoxicity potential of CD8^+^ T cells after culturing in siMALAT1, siHOTAIR, and siMALAT1/siHOTAIR treated TAMs-conditioned media with and without the addition of PDL-1 inhibitor, showing that the cytotoxicity was of higher values upon silencing lncRNAs alone without the addition of the PDL-1 inhibitor.

In conclusion, as shown in Figure 12, HOTAIR is suggested to be an upstream regulator for MALAT1 because upon downregulation of HOTAIR, MALAT1 was also downregulated. Supporting this, downregulation of MALAT1 led to upregulation of HOTAIR as a possible compensatory mechanism explaining that both have the same function. Upon downregulation of MALAT1 and HOTAIR in HER2+ and TNBC, unexpectedly, upregulation of CD80 and MSLN was observed with the fact that silencing HOTAIR was more significant, and upon co-silencing HOTAIR and MALAT1, the expressions of both were upregulated more significantly than silencing each lncRNA, separately.

Moreover, on silencing MALAT1 and HOTAIR in the hormonal subtype, estrogen played an important role due to its effect in downregulation of CD80 and MSLN expressions, thus masking the effect of MALAT1 silencing in upregulating CD80 or MSLN. Certainly, upon co-transfection, the expressions of CD80 and MSLN were upregulated due to the dual action of silencing both lncRNAs, thus dominating the downregulatory activity of estrogen.

It has been concluded that MALAT1 and HOTAIR might be oncogenic lncRNAs in TAMs. This finding was confirmed by the upregulation of VEGF-A protein on silencing MALAT1 and HOTAIR in TAMs of BC and also on assessing the cytotoxicity activity of CD8^+^ T cells on knocking down of these two lncRNAs where the cytotoxicity activity was increased.

Future recommendations involve conducting the dose–response curve for the PDL-1 inhibitor, measuring MSLN and CD80 on the protein level, and examining the interlinkage between MALAT1 and CTLA4 in TAMs of BC.

## Data Availability

The datasets presented in this study can be found in online repositories. The names of the repository/repositories and accession number(s) can be found in the article/Supplementary Material.
