# Supplementary material for: A cutting-edge immunomodulatory interlinkage between HOTAIR and MALAT1 in tumor-associated macrophages in breast cancer: A personalized immunotherapeutic approach
Source: Front Mol Biosci. 2022 Oct 28;9:1032517. doi: 10.3389/fmolb.2022.1032517 (PMC9649622; doi:10.3389/fmolb.2022.1032517)
Supplement: Supplementary file 1 [file Table1.DOCX]

| **Patient** | **Age** | **Histological classification** | **Molecular**  **Classification** | **Pathology**  **Examination**  **(Staging* or**  **Grading**)** | **Mammogram**  **Report***** | **Treatment** |
| --- | --- | --- | --- | --- | --- | --- |
| **Patient 1** | 62 years | Invasive  Lobular  Carcinoma | Luminal A | T1cN3a | BIRADS-5 | No neoadjuvant treatment |
| **Patient 2** | 35 years | Invasive ductal carcinoma | TNBC | Grade II-III | BIRADS-5 | No neoadjuvant treatment |
| **Patient 3** | 58 years | Invasive ductal carcinoma | HER2+ | T2 | BIRADS-5 | Neoadjuvant chemotherapy with  Trastuzumab |
| **Patient 4** | 77 years | Invasive ductal carcinoma | Luminal B | T1  Grade II | BIRADS-5 | No neoadjuvant treatment |
| **Patient 5** | 78 years | Invasive Lobular carcinoma | Luminal B | Grade II | BIRADS-5 | Under  Hormonal  Therapy |
| **Patient 6** | 65 years | Invasive ductal carcinoma | HER2 + | Grade III | BIRADS-5 | Neoadjuvant chemotherapy with  Trastuzumab |
| **Patient 7** | 68 years | Invasive ductal carcinoma | HER2+ | T2N3a  Grade II-III | BIRADS-4c | No neoadjuvant treatment |
| **Patient 8** | 41 years | Invasive ductal carcinoma | TNBC | Grade II-III | BIRADS-4c | No neoadjuvant treatment |
| **Patent 9** | 68 years | Invasive ductal carcinoma | Luminal B | Grade II-III | BIRADS 6 | No neoadjuvant treatment |
| **Patient**  **10** | 36 years | Invasive ductal carcinoma | TNBC | Grade III | BIRADS 6 | Neoadjuvant chemotherapy |
| **Patient**  **11** | 36 years | Invasive ductal carcinoma | HER2+ | Grade II | BIRADS 6 | Neoadjuvant chemotherapy +  Dual AntiHER2  trastuzumab, pertuzumab |
| **Patient**  **12** | 57 years | Invasive ductal carcinoma | Luminal B | Grade II | BIRADS 6 | No neoadjuvant treatment |
| **Patient**  **13** | 60 years | Invasive ductal carcinoma | Luminal A | Grade II-III | BIRADS 6 | No neoadjuvant treatment |
| **Patient**  **14** | 44 years | Invasive ductal carcinoma | Luminal A | Grade II  T2 | BIRADS 6 | No neoadjuvant treatment |
| **Patient**  **15** | 53 years | Invasive ductal carcinoma | Luminal A | Grade II  T1 | BIRADS 5 | No neoadjuvant treatment |
| **Patient**  **16** | 51 years | Invasive ductal carcinoma | TNBC | Grade II-III  T2 | BIRADS 4c | No neoadjuvant treatment |
| **Patient**  **17** | 48 years | Invasive ductal carcinoma | HER2+ | Grade II-III  T1c | BIRADS 6 | No neoadjuvant treatment |
| **Patient**  **18** | 45 years | Invasive ductal carcinoma | Luminal A | Grade II | BIRADS 4b | No neoadjuvant treatment |
| **Patient**  **19** | 70 years | Invasive ductal carcinoma | Luminal A | Grade II  T1b | BIRADS 6 | No neoadjuvant treatment |
| **Patient**  **20** | 75 years | Invasive ductal carcinoma | Luminal A | Grade II | BIRADS 4c | No neoadjuvant treatment |
| **Patient**  **21** | 72 years | Invasive lobular carcinoma | Luminal A | T3N1a | BIRADS 6 | No neoadjuvant treatment |
| **Patient**  **22** | 50 years | Invasive  Ductal  Carcinoma | Luminal B | Grade II | BIRADS 6 | No neoadjuvant treatment |
| **Patient**  **23** | 68 years | Inflammatory | HER2+ | T4N2 | BIRADS 5 | Neoadjuvant chemotherapy |
| **Patient**  **24** | 60 years | Invasive  Ductal  Carcinoma | Luminal A | Grade III | BIRADS 6 | No neoadjuvant treatment |
| **Patient**  **25** | 56 years | Invasive  Ductal  Carcinoma | Luminal A | Grade II-III | BIRADS 6 | No neoadjuvant treatment |
| **Patient**  **26** | 41 years | Invasive  Ductal  Carcinoma | HER2+ | Grade III | BIRADS 6 | Neoadjuvant chemotherapy and Anti-HER2 (Trastuzumab) |
| **Patient**  **27** | 55 years | Invasive  Ductal  Carcinoma | Luminal B | Grade II | BIRADS 6 | Neoadjuvant chemotherapy |
| **Patient**  **28** | 64 years | Invasive  Ductal  Carcinoma | Luminal B | Grade II | BIRADS 6 | No neoadjuvant treatment |
| **Patient**  **29** | 42 years | Invasive  Ductal  Carcinoma | Luminal B | Grade II | BIRADS 4a | Neoadjuvant chemotherapy |

| **Patient**  **30** | 35 years | Invasive  Ductal  Carcinoma | Luminal B | Grade II | BIRADS 4c | No neoadjuvant treatment |
| --- | --- | --- | --- | --- | --- | --- |
| **Patient**  **31** | 40 years | Invasive  Ductal  Carcinoma | Luminal A | Grade II | BIRADS 6 | No neoadjuvant treatment |
| **Patient**  **32** | 46 years | Invasive  Ductal  Carcinoma | Luminal B | Grade II | BIRADS 6 | Neoadjuvant chemotherapy |
| **Patient**  **33** | 34 years | Invasive  Ductal  Carcinoma | Luminal B | Grade II | BIRADS 6 | No neoadjuvant treatment |
| **Patient**  **34** | 52 years | Invasive  Ductal  Carcinoma | Luminal B | Grade II | BIRADS 6 | No neoadjuvant treatment |
| **Patient**  **35** | 73 years | Invasive  Ductal  Carcinoma | TNBC | Grade II | BIRADS 6 | No neoadjuvant treatment |
| **Patient**  **36** | 55 years | Invasive  Ductal  Carcinoma | TNBC | Grade II | BIRADS 6 | No neoadjuvant treatment |
| **Patient**  **37** | 40 years | Invasive  Ductal  Carcinoma | TNBC | Grade II-III | BIRADS 6 | No neoadjuvant treatment |
| **Patient**  **38** | 36 years | Invasive  Ductal  Carcinoma | TNBC | Grade III | BIRADS 6 | No neoadjuvant treatment |
| **Patient**  **39** | 69 years | Invasive  Ductal  Carcinoma | TNBC | Grade III | BIRADS 6 | No neoadjuvant treatment |
| **Patient**  **40** | 46 years | Invasive  Ductal  Carcinoma | TNBC | Grade III | BIRADS 5 | No neoadjuvant treatment |
| **Patient**  **41** | 48 years | Invasive  Ductal  Carcinoma | HER2+ | Grade II | BIRADS 6 | Neoadjuvant chemotherapy with  Trastuzumab |
| **Patient**  **42** | 65 years | Invasive  Ductal  Carcinoma | HER2+ | Grade III | BIRADS 6 | Neoadjuvant chemotherapy with  Trastuzumab |
| **Patient**  **43** | 60 years | Invasive  Ductal  Carcinoma | HER2+ | Grade III | BIRADS 5 | No neoadjuvant treatment |
